# Supplementary material for: ITV-Pal programme: protocol of evaluation of the implementation of tech-volunteer programme in palliative care services
Source: BMJ Open. 2023 Aug 24;13(8):e065222. doi: 10.1136/bmjopen-2022-065222 (PMC10450131; doi:10.1136/bmjopen-2022-065222)
Supplement: Author's manuscript [file bmjopen-2022-065222.draft_revisions.pdf]

BMJ Open is committed to open peer review. As part of this commitment we make the peer review history of every article we publish publicly available.

When an article is published we post the peer reviewers' comments and the authors' responses online. We also post the versions of the paper that were used during peer review. These are the versions that the peer review comments apply to.

The versions of the paper that follow are the versions that were submitted during the peer review process. They are not the versions of record or the final published versions. They should not be cited or distributed as the published version of this manuscript.

BMJ Open is an open access journal and the full, final, typeset and author-corrected version of record of the manuscript is available on our site with no access controls, subscription charges or pay-per-view fees (http://bmjopen.bmj.com).

If you have any questions on BMJ Open's open peer review process please email info.bmjopen@bmj.com

BMJ Open

ITV-Pal Programme: Protocol of evaluation of the Implementation of Tech-Volunteer Programme in Palliative Care Services

Journal:	*BMJ Open*
Manuscript ID	bmjopen-2022-065222
Article Type:	Protocol
Date Submitted by the Author:	08-Jun-2022

Complete List of Authors:	Barnestein-Fonseca, Pilar; CUDECA Foundation; IBIMA,<br>Víbora Martín, Eva; Universidad de Malaga, Department of Social Psychology; CUDECA Foundation<br>Ruiz-Torreras, Inmaculada; CUDECA Foundation; Universidad de Malaga, Department of Social Psychology<br>Chapinal-Bascón, Helena; CUDECA Foundation<br>Martín-Roselló, Maria ; CUDECA Foundation; IBIMA<br>Gómez García, Rafael; CUDECA Foundation; IBIMA

Keywords:	PALLIATIVE CARE, Telemedicine < BIOTECHNOLOGY & BIOINFORMATICS, COVID-19, PUBLIC HEALTH

SCHOLARONE™
Manuscripts

Title: *ITV-Pal Programme: Protocol of evaluation of the Implementation of Tech-Volunteer Programme in Palliative Care Services*

Authors: Pilar Barnestein-Fonseca[2 3] (pilarbarnestein@cudeca.org)
Eva Víbora-Martín [1 2 4] (evavibora@cudeca.org)
Inmaculada Ruiz-Torreras [1 2 4] (inmaruiz@cudeca.org)
Helena Chapinal-Bascón [1] (helenachapinal@cudeca.org)
María Luisa Martín-Roselló [2 4] (marisamartin@cudeca.org)
Rafael Gómez-García [2 4] (rafaelgomez@cudeca.org)

[1] Department of Social Psychology, Social Work, Social Anthropology and East Asian Studies. Faculty of Psychology and Speech Therapy. University of Malaga.

[2] CUDECA Institute for Training and Research in Palliative Care, CUDECA Hospice Foundation, Málaga, Spain.

[3] Instituto de Investigación Biomédica de Málaga-IBIMA Group C08: Pharma economy: Clinical and economic evaluation of medication and Palliative Care, Málaga, Spain.

[4] Instituto de Investigación Biomédica de Málaga-IBIMA Group CA15: Palliative Care, Málaga, Spain.

Correspondence to
Eva Víbora-Martín[1 2 4] (evavibora@cudeca.org)

[1] Department of Social Psychology, Social Work, Social Anthropology and East Asian Studies. Faculty of Psychology and Speech Therapy. University of Malaga.

[2] CUDECA Institute for Training and Research in Palliative Care, CUDECA Hospice Foundation, Málaga, Spain.

[4] Instituto de Investigación Biomédica de Málaga-IBIMA Group CA15: Palliative Care, Málaga, Spain.

+34 652 81 30 16

Abstract

Introduction: Distress at the end of life is significantly associated with physical/somatic and non-physical symptoms but dying is also a social process. Volunteer support represents an important and necessary community resource.

New technologies (NT) may become a useful resource in this kind of situation and that have already proven a benefit in the assistance context of palliative care (PC).

Although volunteers contribute millions of hours of work, the review of bibliography found no sufficiently robust studies to merit inclusion, and even less when associating PC volunteering and NT.

As more research is needed on the impact of training and support for PC volunteers, we intend to study the state of art of technophilia and technophobia, and according to the results, implement and evaluate a volunteer training programme in the use of NT to support patients dying at home and their relatives.

Methods and analysis: we will conduct a pilot study of 20 volunteers and 70 patients over two years. Mix-methods design allows combining quantitative measures of implementation with in-depth

For peer review only

qualitative data.

Ethics and dissemination: Ethics approval for the ITV-Pal Programme project was granted by the Malaga Regional Research Ethics Committee. As new knowledge is gained from this project, findings will be disseminated through publications, presentations and feedback to participating clinics.

Trial registration: NCT04900103

Keywords: New technologies, volunteering, palliative care, digital volunteering.

Article Summary

Strengths and limitations of this study

- The best way to evaluate the implementation of a service is to combine quantitative and qualitative methodology. Therefore, the ITV-PAL programme combines both methods.
- Use quantitative methods to measure key process variables and allow testing of pre-hypothesised mechanisms of impact and contextual moderators.
- Use qualitative methods to capture emerging changes in implementation, experiences of the intervention and unanticipated or complex causal pathways, and to generate new theory.
- It may be that the effect of the intervention on recruitment may result in different recruitment rates. For example, when randomising, a volunteer may not feel comfortable being assigned to the intervention group.
- Principal risk in this study will be that we will not be able to attend the different needs of patients/relatives. In this case, we will try to adapt offering other volunteers who may be able to givea better respond to this need.

Introduction

How we care for the dying is perhaps the most pressing personal, social and public health issue of the 21st century. The experience of dying as an integral and acknowledged part of individual life, supported by the very best personalised care, is currently far from being standard practice, in Europe and beyond. Furthermore, it is estimated that by 2050, 1 in 5 people in the world will be over the age of 60 [1] with an estimated 52 million people expected to die worldwide in 2030 from one of these causes [2]. This poses a new challenge to the functional capacity and organizational structures of current healthcare systems.

Distress at the end of life is significantly associated with both physical/somatic and non-physical symptoms. Pharmacological treatment alone is unlikely to ameliorate all the distress a patient

For peer review only

experiences. When a terminal illness disease breaks into life, it leads to the loss of important relationships, changes the structure of the social network and the levels of relationship and support [2]. Dying is therefore also a social process and psychological distress due to feelings of anxiety, depression, loneliness and being a burden requires additional care approaches. Approaches that aim to provide supplementary, informal, lay support for patients dying at home, and their relatives, may introduce a valuable sense of 'community' and lead to important improvements in care of the dying. Volunteer support represents an important and necessary community resource for patients and relatives to enhance the experience of living at the end of life, and dying although few studies exist, the few that do agree on the beneficial aspects of volunteering on patient and family satisfaction at the end of life [3,4].

The development of innovative, economically viable interventions to support the care that dying patients and their relatives receive is of paramount importance. In this way, the current health emergency situation and the confinement has revealed the isolation and loneliness that certain groups have already lived for a long time, such as the elderly, immigrants or the patients and caregivers [5]. NT may become a useful resource in this kind of situation and that have already proven a benefit in the assistance context of PC. Health care professionals use information and communication technologies to facilitate health services to patients who have problems with the access of the services, any disability which restricts the movement or live in a rural zone [6,7]. In some studies, they found that the use of NTs in consultation was as effective as face-to-face [8-12]. It was even more positive for caregivers, reducing their anxiety and increasing their quality of life [13]. In this context, the implementation of services that combine volunteer support and NT canbe an innovative, economically viable intervention to support the care of dying patients and their relatives.

In addition, these approaches bring volunteering closer to people who would like to offer their help but have little time or cannot move to the place, in these cases the virtual volunteering could be a solution to reach a larger population [14]

The studies related to volunteering with NT mainly address the challenges of bringing these types of devices closer to the older population [15,16] or how volunteers perceive it as another resource to improve their relationship with the caregivers of patients [17], but even so entities are far from including suchresources in their practise.

IT resources facilitate volunteer **interaction and communication** with the community, which is increasingly dependent on NT. Furthermore, volunteers with IT training will be able to help patients with **daily practical tasks.** NT have also demonstrated their utility in **therapeutic interventions,** when patients learn to use them as a tool that may facilitate recovery of self-identity, autobiography, recovery of past memories, the preservation and resolution of interpersonal relationships, the sharing

For peer review only - http://bmjopen.bmj.com/site/about/guidelines.xhtml

of messages to their relatives or the construction of their history for the family and other loved ones [18,19]

Although volunteers contribute millions of hours of work, the review of bibliography found no sufficiently robust studies to merit inclusion, and even less when associating PC volunteering and NT. As more research is needed on the impact of training and support for palliative care volunteers, we intend to study the state of art of technophilia and technophobia among patients, relatives, volunteers and healthcare professionals in PC, and according to the results, implement and evaluate a volunteer training programme in the use of new technology to support patients dying at home and their relatives.

The global **aim of this study is to** implement and evaluate a volunteer training programme in the use of new technology (NT) (specifically through smartphones and tablets) to support patients facing a life threatening illness and their relatives. The specific objectives will be: 1) Explore the need and usefulness of NT from the point of view of patients,relatives, volunteers and health care professionals (HCP) in PC and describe their technological profile; 2) Design a tech-volunteer curriculum and implement a tech- volunteer training programme; 3) Implement the tech-volunteer programme within a PC Home care service and Inpatient unit and assess its impact on the care provided.

Methodology:

Study Design

The best approach to evaluate the implementation process is the combination of quantitative and qualitative methods [20]**.** Basic quantitative measures of implementation may be combined with in-depth qualitative data to provide detailed understandings of intervention functioning on a small scale. Use quantitative methods to measure key process variables and allow testing of pre-hypothesised mechanisms of impact and contextual moderators. Use qualitative methods to capture emerging changes in implementation, experiences of the intervention and unanticipated or complex causal pathways, and to generate new theory.

A - Quantitative approach:

- **Pragmatic cluster randomized clinical trial** to test the efficacy (the unit of randomization is the volunteer and the unit of analysis is the patient/relative). Pragmatic cluster randomised controlled trial has shown to be the most appropriate design when the programmes are more focused on the organisational level. For our study, the cluster design is based on two levels: the higher or second level is represented by the volunteer (over whom the intervention is conducted: ITVPal Programme), and the lower or first level being represented by the patients/relatives, which have accepted to participate and are going to receive the tech-volunteer care from volunteersof ITVPal Programme.

For peer review only - http://bmjopen.bmj.com/site/about/guidelines.xhtml

- **Before-after design** for satisfaction of volunteers and HPC with the intervention and its implementation to test the effectiveness. The design involves evaluating the effects of a deliberate intervention (introduction of ITVPal Programme) by comparing the outcomes of study participants investigated before the intervention with thosemeasured afterwards.
- **Cost-utility study** from the perspective of the funder with a time horizon of two years. A detailed cost analysis including costs for the adaptation of volunteer standard course to ITVPal Programme as well as the recruitment and training costs of the volunteers will be performed.

B - Qualitative approach: **interviews** (individuals and groupal) with HCP, volunteers and with key informants of patients/relatives to test at the beginning the need and usefulness of NT, and also during the implementation process to test changes and experiences. A narrative approach will be adopted, facilitating the production of 'rich' data regarding how participants make 'sense' of and engage with the ITVPal programme. These interviews will explore the thoughts and feelings engendered by access to this service, initial motivations, as well as thoughts and feelings about care delivery as a whole. Narrative interviewing enables the participants to tell their 'stories', prompted byan open question.

Setting

The study will be conducted in a Palliative Care Unit from both Home Care and Inpatient Unit of CUDECA Foundation.

Participants

A total of 140 patients and/or relatives will be selected from Home Care and Inpatient Unit of CUDECA Foundation where the ITV-Pal program will be implemented. This sample size is enough to detect a mean difference of 3 in the HAD scores between groups of 25%, with a power of 80% and a confidence level of 95%. Sample size was adjusted according to the standard criteria for cluster randomized trials, using the design effect (DE) of 1.3. The DE was calculated as follows: DE = 1 + (nc – 1)*ICC (where nc is the mean number of individuals in the cluster and ICC the intra-cluster correlation coefficient). The ICC in the present work was consider to be 0.05 and the mean cluster size was assumed to be 7. A potential loss of 20% was estimated. Therefore, a number of 140 patients/relatives and 20 volunteers were required.

The inclusion criteria will be: patients and families cared for by a specialized Palliative Care team; to be over 18 years of age; consenting to participate in the study by signing the informed consent form. Subjects who have no specific conditions reducing their physical ability to use the devices, for example, visual, hearing, or motor impairments will be excluded from the study.

The HCP involved in these services will be included to measure the implementation success of the program.

For peer review only - http://bmjopen.bmj.com/site/about/guidelines.xhtml

The inclusion criteria for volunteers will be an interview with the Volunteer Department and having completed basic care training to be able to carry out their work. Commitment to participate in the study for one year. For this reason, more volunteers will be trained during the two years of implementation as needed.

Patient and Public Involvement

Not patient involved

Intervention

A complex intervention will be carried out in two phases.

- First phase: training of volunteers in NT. Volunteers will be trained in the use of NT and their interconnectivity as tools to support their work as volunteers.

The topics that will be covered in the volunteer training are: the role of the volunteer, the role of the volunteer in PC, the use of NT (I and II), communication strategies, complicated situations with patients, complicated situations with relatives, self-care, organisation of the unit where they will carry out their volunteering and summary and closure of the training. All of this is divided into 10 sessions. This training will be integrated into the specific training programme for PC care volunteers.

The control group, on the other hand, will receive the standard training that the care volunteers have received so far.

- Second phase: use of NT with patients and relatives. While the control group will accompany patients without using NT, as usual.

Variables

Patients:

- ICECAP-SCM [21]
- EuroQol-5D-5L [22], a generic, brief and easy-to-use questionnaire designed to describe and assess the current health-related quality of life of the person completing the questionnaire. Primary outcome.
- Distress Thermometer [23]
- Edmonton Symptom Assessment Questionnaire [24]
- Tech-PH [25]
- QFSAS [26]
- HADS [27]

For peer review only - http://bmjopen.bmj.com/site/about/guidelines.xhtml
For peer review only

Relatives:

- ICECAP-CPM [28]
- EuroQoL-5D-5L [22]
- Distress Thermometer [23]
- TechPH [25]
- QFSAS [26]
- HADS [27]
- iCODE [29]

Volunteers:

- ICECAP-A [30]
- EuroQoL-5D-5L [22].
- Distress Thermometer [23].
- Satisfaction with volunteering; Motivation (VFI) [31]
- TechPH [25]

HCP:

- TechPH [25]

Focus groups or in-depth interviews will be conducted with all groups at different stages of the study to assess the implementation of the study.

Process Evaluation: Service Improvement Metrics: Process evaluation measures will be collected to assess the ‘success’ of the implementation process and how the service is being delivered. Data will be collected to assess the following: Success of the Infrastructure (fidelity). Whether the intervention was delivered as intended: Referral criteria/identification of patients; Eligibility and take up of the service (dose). The quantity of intervention implemented: Number of patients eligible for the service and number of patients who accept the service; Spread of implementation (reach). Whether the intended audience comes into contact with the intervention, and how: Number of wards with ‘eligible’ patients and number of wards where the service is implemented.

Data collection and follow up

The care teams offer the accompaniment of a volunteer to the patient/family. If they accept the

For peer review only - http://bmjopen.bmj.com/site/about/guidelines.xhtml

For peer review only

accompaniment and participate in the study (in case they want the accompaniment of a volunteer but do not want to participate in the study, the volunteer will be offered anyway). Once the informed consent to participate in the study has been signed, the data collection will proceed as described below. Further details can be seen in figure 1:

Patients/families who consent to participate (intervention and control group) will be invited to fill in at least 3 short questionnaires: at the time of recruitment for this study, 15 days after recruitment following the volunteer service experience (if hospitalised), and every month as long as the patient remains under PC care or until death. During the follow-up, patients and relatives will be asked if there is any change in their condition with respect to the questions asked at baseline. In this way, costly follow-up will be avoided. A maximum of 5 patients/families will also be invited to participate in an in-depth interview at various points during the implementation of the programme.

Volunteers will spend one year accompanying in ITV-Pal. In the following year, there will be other newly trained volunteers joining the project. During their time, they will fill in five questionnaires: baseline, 3, 6 and 12 months following volunteering, and 3 months after. A maximum of 5 volunteers and 5 HCP with direct experienceof the service will be approached for inclusion for in-depth interviews to explore their experiences during the implementation process and at the end of this study.

Figure 1

Schematic of patient enrolment and data collection in the ITV-PAL study.

Data analysis:

Quantitative data: Intention-to-treat principle. Descriptive statistics will be used to summarise subject characteristics (age, gender, education, religion, socioeconomic status, marital status, place of residence) and the process evaluation. Basal comparison between groups for independent samples: Student t-test or chi-square test; for related samples: ANOVA for repeated measures to analyse changes within groups. Multilevelanalysis will be used for main and secondary outcomes. A multilevel modelling approach will be used to examine the difference in the primary endpoint between patients/relatives, taking account of clustering effects at volunteers level. All statistical tests will be two sided and considered significant if$p < 0.05$. Repeated measures analyses of variance will be conducted to assess the development of endpoints over time. Multivariate Imputation will be used to handle missing data.

Also, outcome data will be collected for patients and relatives: the cost-effectiveness of care of dying patients, including the volunteer training programme, will be assessed by calculating the incremental

For peer review only - http://bmjopen.bmj.com/site/about/guidelines.xhtml

cost-effectiveness ratio (ICER). The ICER is defined as the difference in total health care costs in the intervention group (including the costs of performing the intervention) compared to the total costs in the control group, divided by the difference in outcomes. Outcomes will be measured also as QALYs using the EQ-5D health- related quality of life questionnaire. We will also use the capabilities approach (ICECAP). The potential impact of the programme beyond the trial period will be extrapolated using modelling techniques.

Qualitative data: Thematic Analysis, with an iterative approach to coding and analysis. For example, what was said by one participant may inform or shed light on the words of another participant. Analysis will focus on the "substance of the interview" to interpret "meanings and perceptions created and shared during a conversation. Analysis of the data will explore the ideas, assumptions or concepts underpinning what participants are saying. Words or phrases will be coded at latent and semantic level to 'label' meaning identified by those words/phrases. 'Themes' will then be developed from these codes around a 'central observation' or concept to reflect patterns of sharedmeaning. For example, to capture "something important" about how these participants make sense of, and utilise, the ITVPal Programme within the context of end of life care.

Analysis will be conducted within a constructionist paradigm, which asserts that meaning and experience are socially produced. Stages of analysis will be iterative and cyclical rather than linear and analysis may move back and forth between stages. In summary, these principles are:

- Familiarisation and immersion with the data: repeated reading of the interviews
- Generation of initial codes: annotations of initial thoughts against items/categories of text
- Creating themes from codes: The minor items/categories that have initially been identified from the text will then be interrogated to generate overall 'themes'. Engaging with relevant published literature (theoretical perspectives and relevant research) should further enhance the evolving interpretation
- Reviewing themes: ensure all collated extracts of text form a coherent pattern, reorganising and refining themes as required
- Defining and naming themes: to define the 'essence' of what a theme is about
- Reporting the analysis: a narrative 'story' of the data to provide a concise, coherent, logical account.

Cost-utility ratio: this is an exploratory objective (for which a specific design has not been applied). The cost-utility ratio will be estimated by dividing the total cost by the sum of the potential gains expressed in QALYs.

For peer review only

Ethics and Dissemination

The study will be carried out according to the Good Clinical Practice guidelines of the Helsinki declaration[32]. Informed consent is obtained from each patient to take part in the study and to review their clinical records. This project was approved by the corresponding ethics review board of the Malaga Provincial Research Ethics Committee on 25/02/2021.

The database is hosted on the secure REDCap platform at the University of Málaga, Spain. REDCap is an important tool for data access, linkages and mobilisation. On agreeing to participate in the project, clinics receive a random identification code, and the principal investigator and statistics team is blind to the coding.

All results and conclusions drawn from this project will be disseminated through local presentations, national or international meetings, academic publications and feedback to project participants.

In addition, as this project is funded by the La Caixa Foundation, the main results of the study will also be published for dissemination to the general public in different articles uploaded on its website.

Discussion

The coronavirus pandemic is changing the way we are interacting, launching us seemingly overnight from the real world into the virtual world, and seeking ways to address challenges of social distance. Face to face support of palliative care volunteers has temporarily been suspended, so creativity and innovative solutions are needed to continue providing this care and support. The use of ICTs and, specifically, new technologies, allows to rethink the way volunteers act integrating these resources to create "tech-volunteers" that will attend their needs in a different way and also support new needs that have emerged more clearly now due to social distance, with a broader reach. The United NationsOnline Volunteer Programme states: *"Online volunteering is fast, easy – and most of all, effective. When skilled, passionate individuals join forces online with great organizations, everyone wins."* but norobust study has been carried out to support this statement. In this way, this study will bring the real evidence toward NT integration as a useful tool not only to facilitate communication between volunteers and patients/relatives, but also to turn new technologies into instruments that support daily living and enhance care, when in the hands of trained tech-volunteers.

Although this new volunteer programme will be implemented during the pandemic to analyse the effects on patients and their families, we hope that training in new technologies will make it easier for PC volunteers to offer new skills and increase the satisfaction of the people they accompany.

The implementation of this study has some limitations that we will try to control for and take into account. For example, in pragmatic cluster randomised controlled trials, the first bias to take into

For peer review only - http://bmjopen.bmj.com/site/about/guidelines.xhtml

account is the selection bias, as the randomisation is usually applied in the second level, before recruitment of the first-level participants, which is where the intervention impact is measured. However, there are strategies that minimise this bias. First, the selection and follow-up of patients will be carried out by an external researcher who will have no knowledge of volunteer randomisation. Second, the effect of the intervention over recruitment can lead to different recruitment indexes, depending on the groups, due to the possibility that volunteers will be allocated in a group which they do not feel comfortable (e.g avolunteer who does not like the NT -technophobe- and will be allocated in the intervention group) could be less motivated to participate. This issue will be taken into account in the analysis and it will be a result of the implementation process. There could be measurement bias due to mistakes in recording of some variables or interviewer bias due to the administration of the questionnaires. To minimise these biases, interviewers will have been trained previously to ensure that visits will be as homogeneous as possible. A CRF and a manual that explains how each variable is measured will be designed.

Another bias that has already been mentioned is the involvement of volunteers in the year they will collaborate in ITV-Pal. For this reason, more volunteers will be trained. But also the number of patients/volunteers needed to achieve a representative sample and draw conclusions will be difficult to achieve in the two years of piloting the study.

Maybe the principal risk in this study will be that we will not be able to attend the different needs of patients/relatives. In this case, we will try to adapt offering other volunteers who may be able to givea better respond to this need.

Due to in this study we want to assess the implementation of an intervention programme, all these issues that we could find during the process could contribute to improve the programme and would be useful for new research and new intervention implementation in the future.

Acknowledgements We would first of all like to thank Fundación La Caixa for supporting this project and making it possible to make it a reality. We would also like to thank all the patients, families, professionals and volunteers who have participated in the beginning of this project and have allowed this protocol to become a reality.

Authors Contributions All authors have contributed to the development and review of the drafts that were used to submit this project and secure the grant.

EV-M and PB-F have produced the final version of this article and it has been reviewed by the rest of them. All authors have read and approved the final version of the manuscript.

Funding This work was supported by “la Caixa” Foundation (SR20-00841)

Competing interests None declared

ORCID iDs

For peer review only

Pilar Barnestein-Fonseca http://orcid.org/0000-0003-2767-8017

Eva Víbora-Martín http://orcid.org/0000-0003-2680-8019

Inmaculada Ruiz-Torreras https://orcid.org/0000-0001-7155-9627

Helena Chapinal-Bascón http://orcid.org/0000-0001-8783-964X

María Luisa Martín-Roselló https://orcid.org/0000-0002-2206-246X

Rafael Gómez-García https://orcid.org/0000-0003-3904-8733

For peer review only

Supplementary materials

9.- References

[1] Dagli RJ, Sharma A. Polypharmacy: a global risk factor for elderly people. Journal of international oral health: JIOH. 2014, 6(6):i.

[2] Valencia, MIB. Envejecimiento de la población: un reto para la salud pública. Revista Colombiana de Anestesiología. 2012: 40(3), 192-194.

[3] Baron, RS, Cutrona, CE, Hicklin, D, Russel DW, Lubaroff, DM. Social support and immune function among spouses of cáncer patients. En: Hewstone, M, Manstead, A, Stroebe, W (Eds.). The Blackwell reader in social psychology. Oxford: Blackwell; 1997.

[4] Block, EMMD, Casarett, DJ, Spence, C, Gozalo, P, Connor, SR, Teno TM. Got volunteers? Association of hospice use of volunteers with bereaved family members' overall rating of the quality of end-of-life care. J. Pain Symptom Manage. 2010, 39: 502-506.

[5] Herbst-Damm, KL, Kulik JA. Volunteer support, marital status, and the survival times of terminally ill patients. Health Psychol. 2005, 24: 225-229.

[6] Gómez, IFL, Silva, EAT, Correa, CT, Zuluaga, SV, Morales, MMR, Serna, CG, et al. Uso de las tecnologías de información y comunicación para el cuidado del binomio maternofetal: revisión de tema. Medicina U.P.B. 2016; 34: 138-147.

[7] Álvarez-Tobón, VA, Luna-Gómez, IF, Torres-Silva, EA, Higuita-Úsuga, A, Rivera-Mejía, PT. Tecnologías de Información y Comunicación (TIC) aplicadas en cuidados paliativos: revisión de tema. Psicooncología, 2018: 15(2), 345-360.

[8] Rogante, M, Giacomozzi, C, Grigioni, M, et al. Telemedicine in palliative care: a review of systematic reviews. Ann Ist Super Sanita, 2016; 52: 434–442.

[9] Head, BA, Schapmire, TJ, Zheng, Y. Telehealth in palliative care: a systematic review of patient-reported outcomes. J Hosp Palliat Nurs, 2017; 19: 130–139.

[10] Steindal, SA, Nes, AAG, Godskesen, TE, et al. Patients' experiences of telehealth in palliative home care: scoping review. J Med Internet Res, 2020; 22:e16218.

[11] Tricco, AC, Lillie, E, Zarin, W, et al. PRISMA extension for scoping reviews (PRISMA-ScR): checklist and explanation. Ann Intern Med, 2018; 169: 467–473.

[12] Wallace, A, Croucher, K, Quilgars, D, et al. Meeting the challenge: developing systematic reviewing in social policy. Policy Polit, 2004; 32: 455–470.

[13] Demiris, G, Parker Oliver, D, Wittenberg-Lyles, E, et al. A noninferiority trial of a problem-solving intervention for hospice caregivers: in person versus videophone. J Palliat Med, 2012; 15: 653–660.

[14] Butgereit, L. Seven characteristics of a successful virtual volunteering platform, 2011 IST-Africa

For peer review only - http://bmjopen.bmj.com/site/about/guidelines.xhtml

Conference Proceedings, Gaborone, 2011: 1-8.

[15] Mukherjee, D, An Exploratory Study of Older Adults' Engagement with Virtual Volunteerism. Journal of Technology in Human Services. 2010; 28(3): 188-196.

[16] Mukherjee, D, Participation of Older Adults in Virtual Volunteering. Ageing Int. 2011; 36: 253-266.

[17] Campos-Romero, S, Herskovic, V, Fuentes, C, Abarca, E. Perceptions on Connecting Respite Care Volunteers and Caregivers. Int. J. Environ. Res. Public Health 2020, 17: 2911.

[18] Espindola, AV, Benincá, CRS, Scortegagna, SA, Secco, AC, Abreu, APM. Terapia da dignidade para adultos com câncer em cuidados paliativos: um relato de caso. Temas em Psicologia, 2017, 25(2), 733-747.

[19] Frávega, A, Carnino, MA, Dominguez, N, Bernal, C. Adultos Mayores y Nuevas Tecnologías: la superación de una brecha tecnológica. 2008, In III Congreso de Tecnología en Educación y Educación en Tecnología.

[20] Moore, G.F., Audrey S., Barker M., Bond L., Bonel C., Hardeman W., Moore L., O'Cathain A., Tinati T., Wight D., Baird J. Process evaluation of complex interventions: Medical Research Council guidance. BMJ: British Medical Journal, 2015. 350: p. h1258.

[21] Sutton, EJ, Coast, J. Development of a supportive care measure for economic evaluation of end-of-life care using qualitative methods. Palliative medicine, 2014. 28(2): 151-157.

[22] EuroQoL group. EuroQol-a new facility for the measurement of health-related quality of life. Health Policity 1990; 16: 199-208.

[23] Roth, AJ, Kornblith, AB, Batel-Copel, L, Peabody, E, Scher, HI, Holland, JC. Rapid screening for psychologic distress in men with prostate carcinoma: a pilot study. Cancer: Interdisciplinary International Journal of the American Cancer Society, 1998, 82(10): 1904-1908.

[24] Bruera, E, Kuehn, N, Miller, MJ, Selmser, P, Macmillan, K. The Edmonton Symptom Assessment System (ESAS): a simple method for the assessment of palliative care patients. Journal of Palliative Care, 1991, 7: 6-9.

[25] Anderberg, P, Eivazzadeh, S, Berglund, JS. A Novel Instrument for Measuring Older People's Attitudes Towards Technology (TechPH): Development and Validation. Journal of Medical Internet Research, 2019, 21(5): e:13951.

[26] Hombrados-Mendieta, I, Gómez-Jacinto, L, Domínguez-Fuentes, JM, García-Leiva, P, Castro-Travé, M. Types of social support provided by parents, teachers and classmates during adolescence. Journal of Community Psychology, 2012. 40(6): 645–664.

[27] Zigmond, AS, Snaith RP. The hospital anxiety and depression scale. Acta psychiatrica scandinavica, 1983, 67(6):361-70.

[28] Canaway, A, Al-Janabi, H, Kinghorn, P, Bailey, C, Coast, J. Development of a measure (ICECAP-Close Person Measure) through qualitative methods to capture the benefits of end-of-life care to those close to the dying for use in economic evaluation. Palliative Medicine, 2017. 31(1): 53-62.

For peer review only

[29] Mayland, CR, Lees, C, Germain, A, et al. Caring for those who die at home: the use and validation of 'Care Of the Dying Evaluation' (CODE) with bereaved relatives. BMJ Support Palliative Care, 2014. 4: 167–174.

[30] Al-Janabi, H, Flynn, TN, Coast, J. Development of a self-report measure of capability wellbeing for adults: the ICECAP-A. Quality of life research. 2012. 21(1): 167-176.

[31] Clary, EG, Snyder, M, Ridge, RD, Copeland, J, Stukas, AA, Haugen, J, Miene, P. Understanding and assessing the motivations of volunteers: A functional approach. Journal of personality and social psychology. 1998. 74(6): 1516-1530.

[32] World Medical Association. Declaration of Helsinki, Fortaleza Brazil. Paris: World Medical Association; 2013.

10. Figure 1.

Schematic of patient enrolment and data collection in the ITV-PAL study. Own elaboration.

For peer review only

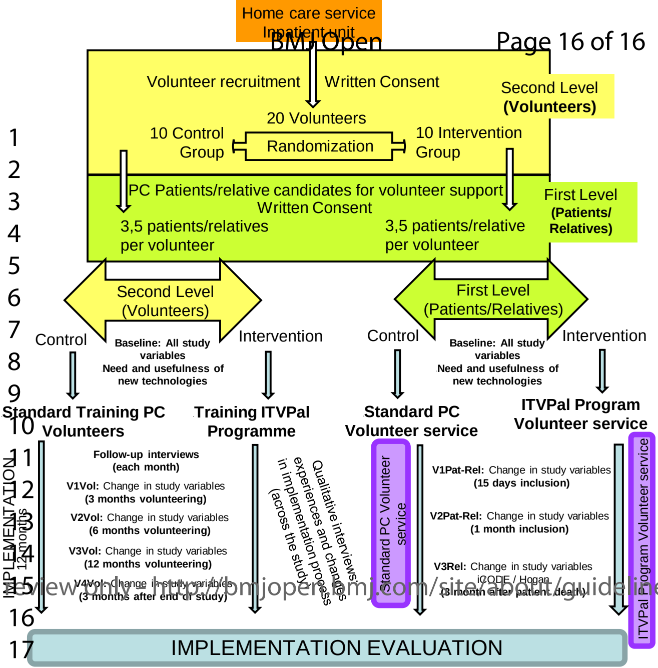

For peer review only - http://bmjopen.bmj.com/site/about/guidelines.xhtml

BMJ Open

ITV-Pal Programme: Protocol of evaluation of the Implementation of Tech-Volunteer Programme in Palliative Care Services

Journal:	*BMJ Open*
Manuscript ID	bmjopen-2022-065222.R1
Article Type:	Protocol
Date Submitted by the Author:	03-Feb-2023
Complete List of Authors:	Barnestein-Fonseca, Pilar; CUDECA Foundation; IBIMA,<br>Víbora Martín, Eva; Universidad de Malaga, Department of Social Psychology; CUDECA Foundation<br>Ruiz-Torreras, Inmaculada; CUDECA Foundation; Universidad de Malaga, Department of Social Psychology<br>Chapinal-Bascón, Helena; CUDECA Foundation<br>Martín-Roselló, Maria ; CUDECA Foundation; IBIMA<br>Gómez García, Rafael; CUDECA Foundation; IBIMA
**Primary Subject Heading**:	Palliative care
Secondary Subject Heading:	Qualitative research
Keywords:	PALLIATIVE CARE, Telemedicine < BIOTECHNOLOGY & BIOINFORMATICS, COVID-19, PUBLIC HEALTH

SCHOLARONE™
Manuscripts

Title: *ITV-Pal Programme: Protocol of evaluation of the Implementation of Tech-Volunteer Programme in Palliative Care Services*

Authors: Pilar Barnestein-Fonseca[2 3] (pilarbarnestein@cudeca.org)
Eva Víbora-Martín [1 2 4] (evavibora@cudeca.org)
Inmaculada Ruiz-Torreras [1 2 4] (inmaruiz@cudeca.org)
Helena Chapinal-Bascón [1] (helenachapinal@cudeca.org)
María Luisa Martín-Roselló [2 4] (marisamartin@cudeca.org)
Rafael Gómez-García [2 4] (rafaelgomez@cudeca.org)

[1] Department of Social Psychology, Social Work, Social Anthropology and East Asian Studies. Faculty of Psychology and Speech Therapy. University of Malaga.

[2] CUDECA Institute for Training and Research in Palliative Care, CUDECA Hospice Foundation, Málaga, Spain.

[3] Instituto de Investigación Biomédica de Málaga-IBIMA Group C08: Pharma economy: Clinical and economic evaluation of medication and Palliative Care, Málaga, Spain.

[4] Instituto de Investigación Biomédica de Málaga-IBIMA Group CA15: Palliative Care, Málaga, Spain.

Correspondence to

Eva Víbora-Martín[1 2 4] (evavibora@cudeca.org)

[1] Department of Social Psychology, Social Work, Social Anthropology and East Asian Studies. Faculty of Psychology and Speech Therapy. University of Malaga.

[2] CUDECA Institute for Training and Research in Palliative Care, CUDECA Hospice Foundation, Málaga, Spain.

[4] Instituto de Investigación Biomédica de Málaga-IBIMA Group CA15: Palliative Care, Málaga, Spain.

+34 652 81 30 16

Abstract

Introduction: Distress at the end of life is significantly associated with physical/somatic and non-physical symptoms, but dying is also a social process. Volunteer support represents an important and necessary community resource.

New technologies (NT) may become a useful resource in this kind of situation and have already proven a benefit in the assistance context of palliative care (PC).

Although volunteers contribute millions of hours of work, the review of bibliography found no sufficiently robust studies to merit inclusion, and even less when associating PC volunteering and NT.

As more research is needed on the impact of training and support for PC volunteers, we intend to study the state of art of technophilia and technophobia, and according to the results, implement and evaluate a volunteer training programme in the use of NT to support patients dying at home and their relatives.

Methods and analysis: we will conduct a pilot study of 20 volunteers and 70 patients over two years. Mix-methods design allows combining quantitative measures of implementation with in-depth

For peer review only

qualitative data.

Ethics and dissemination: Ethics approval for the ITV-Pal Programme project was granted by the Malaga Regional Research Ethics Committee. As new knowledge is gained from this project, findings will be disseminated through publications, presentations and feedback to participating clinics.

Trial registration: NCT04900103

Keywords: New technologies, volunteering, palliative care, digital volunteering.

Article Summary

Strengths and limitations of this study

- The best way to evaluate the implementation of a service is to combine quantitative and qualitative methodology. Therefore, the ITV-PAL programme combines both methods. Quantitative methods are used to measure key process variables and allow testing of pre-hypothesised mechanisms of impact and contextual moderators. Qualitative methods help to capture emerging changes in implementation, experiences of the intervention and unanticipated or complex causal pathways, as well as to generate new theory.
- It may be that the effect of the intervention on recruitment results in different recruitment rates. For example, when randomising, a volunteer may not feel comfortable being assigned to the intervention group.
- The principal risk in this study is that we may not be able to attend to the different needs of patients/relatives. In this case, we will try to adapt by offering other volunteers who may be able to give a better response to these needs.

Introduction

How we care for the dying is perhaps the most pressing personal, social and public health issue of the 21st century. The experience of dying as an integral and acknowledged part of individual life, supported by the very best personalised care, is currently far from being standard practice in Europe and beyond [1]. Furthermore, it is estimated that by 2050, 1 in 5 people in the world will be over the age of 60 [1] with an estimated 52 million people expected to die worldwide in 2030 from chronic diseases such as cancer, cardiovascular and renal diseases and others [2]. This poses a new challenge to the functional capacity and organisational structures of current healthcare systems.

Distress at the end of life is significantly associated with both physical/somatic and non-physical symptoms. For this reason, patient care cannot focus only on treating physical symptoms through

For peer review only

medication. When a terminal illness disrupts a life, it leads to the loss of important relationships, as well as changing the structure of the patient's social network and the levels of relationship and support [2]. Dying is therefore also a social process. The psychological distress due to feelings of anxiety, depression, loneliness and being a burden requires additional approaches to care [3, 4]. Approaches that aim to provide supplementary, informal, lay support for patients dying at home, and their relatives, may introduce a valuable sense of 'community' and lead to important improvements in the care of the dying. Volunteer support represents an important and necessary community resource for patients and relatives to enhance the experience of living at the end of life and dying. Although few studies exist, those few agree on the beneficial aspects of volunteering on patient and family satisfaction at the end of life [3,4].

It is of paramount importance to develop innovative, economically viable interventions to support the care that dying patients and their relatives receive. In this way, the current health emergency situation and the confinement has revealed the isolation and loneliness that certain groups have already experienced for a long time, such as the elderly, immigrants or patients and caregivers [5]. NT may become a useful resource in this kind of situation and have already proven a benefit in the context of PC. Health care professionals use information and communication technologies to facilitate health services for patients who have problems with access to the services, any disability which restricts movement or who live in a rural area [6,7]. In some studies, they found that the use of NT in consultation was as effective as face-to-face [8-12]. It was even more positive for caregivers, reducing their anxiety and increasing their quality of life [13]. In this context, the implementation of services that combine volunteer support and NT can be an innovative, economically viable intervention to support the care of dying patients and their relatives. In addition, these approaches bring volunteering closer to people who would like to offer their help but have little time or cannot be in the same place as the patient/relatives. In these cases virtual volunteering could be a way to reach a larger population [14].

Studies related to volunteering with NT mainly address the challenges of bringing these types of devices closer to the older population [15,16] or how volunteers perceive them as another resource to improve their relationship with the caregivers of patients [17], but even so organisations are far from including such resources in their practice.

IT resources facilitate volunteer **interaction and communication** with the community, which is increasingly dependent on NT. Furthermore, volunteers with IT training will be able to help patients with **daily practical tasks.** NTs have also proven useful for **therapeutic interventions.** The patient can use them to review their biography, maintain social relationships with people who live far away or help them to build a legacy for their loved ones. [18,19]

For peer review only

Although volunteers contribute many hours to accompanying and supporting patients and their relatives, the review of bibliography found no sufficiently robust studies to merit inclusion, and even less when associating PC volunteering and NT. More research is needed to assess the impact of training and support of volunteers in PC. This study aims to analyse the technophilia and technophobia of patients, relatives, volunteers and health professionals. Once their knowledge of new technology has been assessed, a training programme for volunteers will be evaluated and a programme of accompaniment through technological devices will be implemented.

The global **aim of this study is to** evaluate the implementation of a volunteer training programme in the use of NT (specifically through smartphones and tablets) to support patients facing a life-threatening illness and their relatives. The specific objectives will be: 1) To explore the need and usefulness of NT from the point of view of patients, relatives, volunteers and health care professionals (HCP) in PC and describe their technological profile; 2) To design a tech-volunteer curriculum and implement a tech-volunteer training programme; 3) To implement the tech-volunteer programme within a PC Home care service and Inpatient unit and assess its impact on the care provided.

For peer review only

Methodology:

The Research Ethical Committee of Málaga (25/02/2021) approved this study.

Study Design

The best approach to evaluate the implementation process is the combination of quantitative and qualitative methods [20]**.** Basic quantitative measures of implementation may be combined with in-depth qualitative data to provide detailed understanding of intervention functioning on a small scale. Quantitative methods will measure key process variables and allow testing of pre-hypothesised mechanisms of impact and contextual moderators. Qualitative methods will capture emerging changes in implementation, experiences of the intervention and unanticipated or complex causal pathways, as well as generating a new theory.

A - Quantitative approach:

- **Pragmatic cluster randomised clinical trial** to test efficacy (the unit of randomization is the volunteer and the unit of analysis is the patient/relatives). A pragmatic cluster randomised controlled trial has shown to be the most appropriate design when the programmes are more focused on the organisational level [21,22]. For our study, the cluster design is based on two levels: the higher or second level is represented by the volunteer (over volunteer the intervention is conducted: ITVPal Programme), and the lower or first level being represented by the patients/relatives, who have agreed to participate and are going to receive the tech-volunteer care from volunteers of ITVPal Programme.
- **Before-after design** for satisfaction of volunteers and HPC with the intervention and its

implementation to test effectiveness. The design involves evaluating the effects of a deliberate intervention (introduction of ITVPal Programme) by comparing the outcomes of study participants investigated before the intervention with those measured afterwards.

- **Cost-utility study** from the perspective of the funder with a time horizon of two years. A detailed cost analysis including costs for the adaptation of a volunteer standard course to an ITVPal Programme as well as the recruitment and training costs of the volunteers will be performed.

B - Qualitative approach: **interviews** (individuals and groups) with HCP, volunteers and with key informants of patients/relatives to test at the beginning the need and usefulness of NT, and also during the implementation process to test changes and experiences. A narrative approach will be adopted, facilitating the production of 'rich' data regarding how participants make 'sense' of and engage with the ITVPal programme. These interviews will explore the thoughts and feelings engendered by access to this service, initial motivations, as well as thoughts and feelings about care delivery as a whole. Narrative interviewing enables the participants to tell their 'stories', prompted by an open question.

Settings

The study will be conducted in a Palliative Care Unit from both a Home Care and an Inpatient Unit of the CUDECA Foundation.

The Hospitalisation Unit is a small Hospice consisting of 10 rooms where patients at the end of life are cared for in cases of uncontrolled symptoms, for family respite or in their final days, among other situations. In this unit, volunteers come every afternoon to the rooms where they wish to offer accompaniment to patients and their relatives.

In addition, the foundation also provides home care to more than 1,500 patients a year through 7 care teams made up of a doctor, nurse, psychologist and social worker. In these cases, the volunteer accompanies patients and relatives who wish them to do so.

Participants

A total of 140 patients and/or relatives will be selected from the Home Care and Inpatient Unit of CUDECA Foundation where the ITV-Pal program will be implemented. This sample size is enough to detect a mean difference of 3 in the HAD scores between groups of 25%, with a power of 80% and a confidence level of 95% [23]. Sample size was adjusted according to the standard criteria for cluster randomised trials, using the design effect (DE) of 1.3. The DE was calculated as follows: DE = 1 + (nc – 1)*ICC (where nc is the mean number of individuals in the cluster and ICC the intra-cluster correlation coefficient). The ICC in the present work was considered to be 0.05 and the mean cluster size was assumed to be 7. A potential loss of 20% was estimated. Therefore, 140 patients/relatives and 20

For peer review only

volunteers are required.

The inclusion criteria will be: patients and relatives cared for by a specialised Palliative Care team; to be over 18 years of age; consenting to participate in the study by signing the informed consent form. Subjects who have specific conditions reducing their physical ability to use the devices, for example, visual, hearing, or motor impairments will be excluded from the study.

The HCP involved in these services will be included to measure the success of implementing the program.

The inclusion criteria for volunteers will be an interview with the Volunteer Department and having completed basic care training to be able to carry out their work. Also a commitment to participate in the study for one year. For this reason, more volunteers will be trained as needed during the two years of implementation.

Patient and Public Involvement

No patient involved

Intervention

A complex intervention will be carried out in two phases.

- First phase: training of volunteers in NT. Volunteers will be trained in the use of NT and their interconnectivity as tools to support their work as volunteers.

The topics that will be covered in the volunteer training are the role of the volunteer, use of NT, communication strategies, complicated situations, self-care, summary and closure of the training. All of this is divided into 10 sessions. This training will be integrated into the specific training programme for PC care volunteers.

The control group, on the other hand, will receive the standard training that the care volunteers have received so far.

- Second phase: volunteer accompaniment of patients/relatives. During the activities carried out by the volunteer with the patient for accompaniment and leisure. It will have the support of a technological device to be used according to the needs of the patient/relatives (e.g. internet searches on a topic of interest, video calls, phone calls, virtual visits, etc.) It will be an outcome of the study to know what the device has been used for.

The control group will accompany patients without using the NT as usual.

For peer review only

Variables

Sociodemographic variables such as date of birth, gender, educational level, nationality, spiritual beliefs and employment status will be assessed in all groups.

Specifically, relatives will be asked about their relationship with the patient. Volunteers will be questioned about their time of collaboration and whether they have had personal experience with palliative patients and finally, health professionals will be asked specifically about their work experience in Palliative Care.

In the following section, the different tools of assessment that each group will have to complete will be indicated:

Patients:

- ICECAP-SCM [24] has been developed as a tool for use in economic evaluation conducted in an end of life setting for patients. The questionnaire covers the attributes of: choice, love and affection, physical suffering, emotional suffering, dignity, being supported and preparation with the lowest score being 1 and the highest score being 4.

- EuroQol-5D-5L [25], a generic, brief and easy-to-use questionnaire designed to describe and assess the current health-related quality of life of the person completing the questionnaire. Primary outcome. The descriptive system comprises five dimensions: mobility, self-care, usual activities, pain/discomfort and anxiety/depression. Each dimension has 5 levels: no problems, slight problems, moderate problems, severe problems and extreme problems. The patient is asked to indicate his/her health state by ticking the box next to the most appropriate statement in each of the five dimensions.

- Distress Thermometer [26] was developed as a simple tool to effectively screen for symptoms of distress. The instrument is a self-reported tool using a 0-to-10 rating scale.

- Edmonton Symptom Assessment Questionnaire [27] used to rate the intensity of nine common symptoms experienced by cancer patients, including pain, tiredness, nausea, depression, anxiety, drowsiness, appetite, well-being and shortness of breath where 0 is that the symptomatology does not affect him/her at the moment and 10 with the highest intensity.

- Tech-PH [28] an instrument for measuring older people's attitudes toward technology. It was created from the six items in the two factors, techEnthusiasm and techAnxiety. TechPH could be interpreted on a five-point response scale, ranging from 1 (fully disagree) to 5 (fully agree), where the higher the index indicates a higher level of technophilia.

- QFSAS [29] was designed to assess the frequency and degree of satisfaction with perceived

For peer review only

social support from different sources in relation to three types of support: emotional, informational and instrumental. Both frequency and satisfaction are measured on a likert scale of 1 to 5.

- HADS [30] instrument to measure anxiety and depression in a general medical population of patients. The lowest score being 0 and the highest score being 3.

Relatives:

- ICECAP-CPM [31] has been developed as a tool for use in economic evaluation conducted in an end of life setting for relatives. The questionnaire covers the attributes of: good communication, privacy and space, emotional support, practical support, being able to prepare and cope and being free from emotional distress related to the condition of the decent with the lowest score being 1 and the highest score being 5.
- EuroQoL-5D-5L [25]
- Distress Thermometer [26]
- TechPH [28]
- QFSAS [29]
- HADS [30]
- iCODE [32] is a self-completion post-bereavement questionnaire, based on the key components of best practice in end of life care. It assesses quality of care for the dying through 32 main questions, reflecting core palliative care principles.

Volunteers:

- ICECAP-A [33] is a measure of capability for the general adult (18+) population for use in economic evaluation. The questionnaire covers the attributes of: attachment (an ability to have love, friendship and support), stability (an ability to feel settled and secure), achievement (an ability to achieve and progress in life), enjoyment (an ability to experience enjoyment and pleasure) and autonomy (an ability to be independent) with the lowest score being 1 and the highest score being 4.
- EuroQoL-5D-5L [25].
- Distress Thermometer [26].
- Satisfaction with volunteering; Motivation (VFI) [34] a 30 item measure of motivations to volunteer. Respondents answer each item on a 7-point scale ranging from 1 (not at all

For peer review only

important/accurate) to 7 (extremely important/accurate).

- TechPH [28]

HCP:

- TechPH [28]

Focus groups or in-depth interviews will be conducted with all groups at different stages of the study to assess the implementation of the study.

Process Evaluation: Service Improvement Metrics: Process evaluation measures will be collected to assess the 'success' of the implementation process and how the service is being delivered. Data will be collected to assess the following: fidelity, dose and reach. Fidelity: Success of the Infrastructure. Dose: identification of patients, eligibility and take up of the service and whether the intervention was delivered as intended. Reach: (spread of implementation) quantifying the intervention implemented with the number of patients eligible for the service, number of patients who accept the service, number of wards with 'eligible' patients and number of wards where the service is implemented.

Data collection and follow up

The recruitment of the volunteers began in March 2021. When they finished their training, the new service started in September 2021 which was when patients/relatives recruitment started. Volunteer recruitment will be active during the whole study every three months. Recruitment is expected to end in September 2023.

The care teams offer the accompaniment of a volunteer to the patient/relatives. If they accept the accompaniment and participate in the study (if they want the accompaniment of a volunteer but do not want to participate in the study, the volunteer will be offered anyway). Once the informed consent to participate in the study has been signed, the data collection will proceed as described below. Further details can be seen in figure 1:

Patients/relatives who consent to participate (intervention and control group) will be invited to fill in at least 3 short questionnaires: at the time of recruitment for this study, 15 days after recruitment following the volunteer service experience (if hospitalised), and every month as long as the patient remains under PC care or until death. During the follow-up, patients and relatives will be asked if there is any change in their condition with respect to the questions asked at baseline. In this way, costly follow-up will be avoided. A maximum of 5 patients/relatives will also be invited to participate in an in-depth interview at various points during the implementation of the programme.

Volunteers will spend one year accompanying patients in ITV-Pal. In the following year, there will be

For peer review only

other newly trained volunteers joining the project. During their time, they will fill in five questionnaires: baseline, 3, 6 and 12 months following volunteering, and 3 months after. A maximum of 5 volunteers and 5 HCP with direct experience of the service will be approached for inclusion in in-depth interviews to explore their experiences during the implementation process and at the end of this study.

Figure 1

Schematic of patient enrolment and data collection in the ITV-PAL study. Own elaboration.

For peer review only

Data analysis:

Quantitative data: Intention-to-treat principle. Descriptive statistics will be used to summarise subject characteristics (age, gender, education, religion, socioeconomic status, marital status, place of residence) and the process evaluation. Basal comparison between groups for independent samples: Student t-test or chi-square test; for related samples: ANOVA for repeated measures to analyse changes within groups. Multilevel analysis will be used for main and secondary outcomes. A multilevel modelling approach will be used to examine the difference in the primary endpoint between patients/relatives, taking account of clustering effects at volunteers level. All statistical tests will be two sided and considered significant if $p < 0.05$. Repeated measurements of analyses of variance will be conducted to assess the development of endpoints over time. Multivariate Imputation will be used to handle missing data.

Also, outcome data will be collected for patients and relatives: the cost-effectiveness of care of dying patients, including the volunteer training programme, will be assessed by calculating the incremental cost-effectiveness ratio (ICER). The ICER is defined as the difference in total health care costs in the intervention group (including the costs of performing the intervention) compared to the total costs in the control group, divided by the difference in outcomes [35]. Outcomes will be measured also as QALYs using the EQ-5D health- related quality of life questionnaire. We will also use the capabilities approach (ICECAP). The potential impact of the programme beyond the trial period will be extrapolated using modelling techniques.

Qualitative data: Thematic Analysis, with an iterative approach to coding and analysis. For example, what was said by one participant may inform or shed light on the words of another participant. Analysis will focus on the “substance of the interview” to interpret “meanings and perceptions created and shared during a conversation. Analysis of the data will explore the ideas, assumptions or concepts underpinning what participants are saying. Words or phrases will be coded at latent and semantic

level to 'label' meaning identified by those words/phrases. 'Themes' will then be developed from these codes around a 'central observation' or concept to reflect patterns of shared meaning [36]. For example, to capture "something important" about how these participants make sense of, and utilise, the ITVPal Programme within the context of end of life care.

Analysis will be conducted within a constructionist paradigm, which asserts that meaning and experience are socially produced. Stages of analysis will be iterative and cyclical rather than linear and analysis may move back and forth between stages. In summary, these principles are [35]:

- Familiarisation and immersion with the data: repeated reading of the interviews.
- Generation of initial codes: annotations of initial thoughts against items/categories of text.
- Creating themes from codes: The minor items/categories that have initially been identified from the text will then be interrogated to generate overall 'themes'. Engaging with relevant published literature (theoretical perspectives and relevant research) should further enhance the evolving interpretation.
- Reviewing themes: ensure all collated extracts of text form a coherent pattern, reorganising and refining themes as required.
- Defining and naming themes: to define the 'essence' of what a theme is about.
- Reporting the analysis: a narrative 'story' of the data to provide a concise, coherent, logical account.

Cost-utility ratio: this is an exploratory objective (for which a specific design has not been applied). The cost-utility ratio will be estimated by dividing the total cost by the sum of the potential gains expressed in QALYs.

Ethics and Dissemination

The study will be carried out according to the Good Clinical Practice guidelines of the Helsinki declaration [37]. Informed consent is obtained from each patient to take part in the study and for their clinical records to be reviewed. This project was approved by the corresponding ethics review board of the Malaga Provincial Research Ethics Committee on 25/02/2021.

The database is hosted on the secure REDCap platform at the University of Málaga, Spain. REDCap is an important tool for data access, linkages and mobilisation. On agreeing to participate in the project, clinics receive a random identification code, and the principal investigator and statistics team is blind to the coding.

All results and conclusions drawn from this project will be disseminated through local presentations, national or international meetings, academic publications and feedback to project participants.

In addition, as this project is funded by the La Caixa Foundation, the main results of the study will also be published for dissemination to the general public in different articles uploaded on its website.

Discussion

The coronavirus pandemic is changing the way we are interacting, launching us seemingly overnight from the real world into the virtual world, and seeking ways to address the challenges of social distance [38]. Face to face support of palliative care volunteers has temporarily been suspended, so creativity and innovative solutions are needed to continue providing this care and support. The use of ICTs and, specifically, NT, allows us to rethink the way volunteers act by integrating these resources to create "tech-volunteers" that will attend to their needs in a different way and also support new needs that have emerged more clearly now due to social distance, with a broader reach [39]. The United Nations Online Volunteer Programme states: *"Online volunteering is fast, easy – and most of all, effective when skilled, passionate individuals join forces online with great organisations, everyone wins."* but no robust study has been carried out to support this statement. In this way, this study will bring the real evidence toward NT integration as a useful tool not only to facilitate communication between volunteers and patients/relatives, but also to turn NT into instruments that support daily living and enhance care, when in the hands of trained tech-volunteers.

Although this new volunteer programme will be implemented during the pandemic to analyse the effects on patients and their relatives, we hope that training in new technologies will make it easier for PC volunteers to offer new skills and increase the satisfaction of the people they accompany.

The implementation of this study has some limitations that we will try to control for and take into account. For example, in pragmatic cluster randomised controlled trials, the first bias to take into account is the selection bias, as the randomisation is usually applied in the second level, before recruitment of the first-level participants, which is where the intervention impact is measured. However, there are strategies that minimise this bias. First, the selection and follow-up of patients will be carried out by an external researcher who will have no knowledge of volunteer randomisation. Second, the effect of the intervention over recruitment can lead to different recruitment indexes, depending on the groups, due to the possibility that volunteers will be allocated a group in which they do not feel comfortable (e.g a volunteer who does not like the NT -technophobe- and will be allocated the intervention group) could be less motivated to participate. This issue will be taken into account in the analysis and it will be a result of the implementation process. There could be measurement bias due to mistakes in recording of some variables or interviewer bias due to the administration of the questionnaires. To minimise these biases, interviewers will have been trained previously to ensure that visits will be as homogeneous as possible. A data collection booklet and a manual that explains how each variable is measured will be designed.

Another bias that has already been mentioned is the involvement of volunteers in the year they will collaborate in ITV-Pal. For this reason, more volunteers will be trained. But also the number of patients/volunteers needed to achieve a representative sample and draw conclusions will be difficult to

For peer review only

achieve in the two years of piloting the study.

Maybe the principal risk in this study will be that we will not be able to attend to the different needs of patients/relatives. In this case, we will try to adapt by offering other volunteers who may be able to give a better response to this need.

Due to the fact that in this study we want to assess the implementation of an intervention programme, all these issues that we might find during the process could contribute to an improvement of the programme and would be useful for new research and the implementation of new interventions in the future.

Acknowledgements We would first of all like to thank Fundación La Caixa for supporting this project and for enabling us to make it a reality. We would also like to thank all the patients, relatives, professionals and volunteers who have participated in the beginning of this project and have allowed this protocol to become a reality.

Authors Contributions

EV-M, PB-F and IR-T were in charge of project conception and protocol writing. PB-F focused on the methodological part and EV-M and IR-T on the other parts. MLM-R revised the protocol for its final version.

EV-M and PB-F have produced the final version of this article and it has been reviewed by the rest of them. All authors have read and approved the final version of the manuscript.

All authors (EV-M, PB-F, IR-T, MLM-R, RG-G and HC-B)have contributed to the development and review of the drafts that were used to submit this project and secure the grant.

Funding This work was supported by “la Caixa” Foundation (SR20-00841)

Competing interests None declared

ORCID iDs

Pilar Barnestein-Fonseca http://orcid.org/0000-0003-2767-8017

Eva Víbora-Martín http://orcid.org/0000-0003-2680-8019

Inmaculada Ruiz-Torreras https://orcid.org/0000-0001-7155-9627

Helena Chapinal-Bascón http://orcid.org/0000-0001-8783-964X

María Luisa Martín-Roselló https://orcid.org/0000-0002-2206-246X

Rafael Gómez-García https://orcid.org/0000-0003-3904-8733

For peer review only

References

[1] Dagli RJ, Sharma A. Polypharmacy: a global risk factor for elderly people. Journal of international oral health: JIOH. 2014, 6(6):i.

[2] Valencia, MIB. Envejecimiento de la población: un reto para la salud pública. Revista Colombiana de Anestesiología. 2012: 40(3), 192-194.

[3] Baron, RS, Cutrona, CE, Hicklin, D, Russel DW, Lubaroff, DM. Social support and immune function among spouses of cáncer patients. En: Hewstone, M, Manstead, A, Stroebe, W (Eds.). The Blackwell reader in social psychology. Oxford: Blackwell; 1997.

[4] Block, EMMD, Casarett, DJ, Spence, C, Gozalo, P, Connor, SR, Teno TM. Got volunteers? Association of hospice use of volunteers with bereaved family members' overall rating of the quality of end-of-life care. J. Pain Symptom Manage. 2010, 39: 502-506.

[5] Herbst-Damm, KL, Kulik JA. Volunteer support, marital status, and the survival times of terminally ill patients. Health Psychol. 2005, 24: 225-229.

[6] Gómez, IFL, Silva, EAT, Correa, CT, Zuluaga, SV, Morales, MMR, Serna, CG, et al. Uso de las tecnologías de información y comunicación para el cuidado del binomio maternofetal: revisión de tema. Medicina U.P.B. 2016; 34: 138-147.

[7] Álvarez-Tobón, VA, Luna-Gómez, IF, Torres-Silva, EA, Higuita-Úsuga, A, Rivera-Mejía, PT. Tecnologías de Información y Comunicación (TIC) aplicadas en cuidados paliativos: revisión de tema. Psicooncología, 2018: 15(2), 345-360.

[8] Rogante, M, Giacomozzi, C, Grigioni, M, et al. Telemedicine in palliative care: a review of systematic reviews. Ann Ist Super Sanita, 2016; 52: 434–442.

[9] Head, BA, Schapmire, TJ, Zheng, Y. Telehealth in palliative care: a systematic review of patient-reported outcomes. J Hosp Palliat Nurs, 2017; 19: 130–139.

[10] Steindal, SA, Nes, AAG, Godskesen, TE, et al. Patients' experiences of telehealth in palliative home care: scoping review. J Med Internet Res, 2020; 22:e16218.

[11] Tricco, AC, Lillie, E, Zarin, W, et al. PRISMA extension for scoping reviews (PRISMA-ScR): checklist and explanation. Ann Intern Med, 2018; 169: 467–473.

[12] Wallace, A, Croucher, K, Quilgars, D, et al. Meeting the challenge: developing systematic reviewing in social policy. Policy Polit, 2004; 32: 455–470.

[13] Demiris, G, Parker Oliver, D, Wittenberg-Lyles, E, et al. A noninferiority trial of a problem-solving intervention for hospice caregivers: in person versus videophone. J Palliat Med, 2012; 15: 653–660.

[14] Butgereit, L. Seven characteristics of a successful virtual volunteering platform, 2011 IST-Africa

For peer review only

Conference Proceedings, Gaborone, 2011: 1-8.

[15] Mukherjee, D, An Exploratory Study of Older Adults' Engagement with Virtual Volunteerism. Journal of Technology in Human Services. 2010; 28(3): 188-196.

[16] Mukherjee, D, Participation of Older Adults in Virtual Volunteering. Ageing Int. 2011; 36: 253-266.

[17] Campos-Romero, S, Herskovic, V, Fuentes, C, Abarca, E. Perceptions on Connecting Respite Care Volunteers and Caregivers. Int. J. Environ. Res. Public Health 2020, 17: 2911.

[18] Espindola, AV, Benincá, CRS, Scortegagna, SA, Secco, AC, Abreu, APM. Terapia da dignidade para adultos com câncer em cuidados paliativos: um relato de caso. Temas em Psicologia, 2017, 25(2), 733-747.

[19] Frávega, A, Carnino, MA, Dominguez, N, Bernal, C. Adultos Mayores y Nuevas Tecnologías: la superación de una brecha tecnológica. 2008, In III Congreso de Tecnología en Educación y Educación en Tecnología.

[20] Moore, G.F., Audrey, S., Barker, M., Bond, L., Bonel, C., Hardeman, W., Moore, L., O'Cathain, A., Tinati, T., Wight, D., Baird, J. Process evaluation of complex interventions: Medical Research Council guidance. BMJ: British Medical Journal, 2015. 350: p. h1258.

[21] Campbell, M.J. Cluster randomised trials. Med J Aust, 2019, 210, (4), 154-156

[22] Campbell, M.J., Hemming, K., Taljaard, M. The stepped wedge cluster randomised trial: what it is and when it should be used. Med J Aust, 2019, 210, (6), 253-254.

[23] Bernhard, J, Sullivan, M, Hürny, C, Coates, A.S., Rudenstam, C.M. Clinical relevance of single item quality of life indicators in cancer clinical trials. British journal of cancer, 2001. 84(9): 1156-1165.

[24] Sutton, E.J., Coast, J. Development of a supportive care measure for economic evaluation of end-of-life care using qualitative methods. Palliative medicine, 2014. 28(2): 151-157.

[25] EuroQoL group. EuroQol-a new facility for the measurement of health-related quality of life. Health Policy 1990; 16: 199-208.

[26] Roth, AJ, Kornblith, AB, Batel-Copel, L, Peabody, E, Scher, HI, Holland, JC. Rapid screening for psychologic distress in men with prostate carcinoma: a pilot study. Cancer: Interdisciplinary International Journal of the American Cancer Society, 1998, 82(10): 1904-1908.

[27] Bruera, E, Kuehn, N, Miller, MJ, Selmser, P, Macmillan, K. The Edmonton Symptom Assessment System (ESAS): a simple method for the assessment of palliative care patients. Journal of Palliative Care, 1991, 7: 6-9.

[28] Anderberg, P, Eivazzadeh, S, Berglund, JS. A Novel Instrument for Measuring Older People's Attitudes Towards Technology (TechPH): Development and Validation. Journal of Medical Internet Research, 2019, 21(5): e:13951.

[29] Hombrados-Mendieta, I, Gómez-Jacinto, L, Domínguez-Fuentes, JM, García-Leiva, P, Castro-Travé, M. Types of social support provided by parents, teachers and classmates during adolescence. Journal of Community Psychology, 2012. 40(6): 645–664.

For peer review only

[30] Zigmond, AS, Snaith RP. The hospital anxiety and depression scale. Acta psychiatrica scandinavica, 1983, 67(6):361-70.

[31] Canaway, A, Al-Janabi, H, Kinghorn, P, Bailey, C, Coast, J. Development of a measure (ICECAP-Close Person Measure) through qualitative methods to capture the benefits of end-of-life care to those close to the dying for use in economic evaluation. Palliative Medicine, 2017. 31(1): 53-62.

[32] Mayland, CR, Lees, C, Germain, A, et al. Caring for those who die at home: the use and validation of 'Care Of the Dying Evaluation' (CODE) with bereaved relatives. BMJ Support Palliative Care, 2014. 4: 167–174.

[33] Al-Janabi, H, Flynn, TN, Coast, J. Development of a self-report measure of capability wellbeing for adults: the ICECAP-A. Quality of life research. 2012. 21(1): 167-176.

[34] Clary, EG, Snyder, M, Ridge, RD, Copeland, J, Stukas, AA, Haugen, J, Miene, P. Understanding and assessing the motivations of volunteers: A functional approach. Journal of personality and social psychology. 1998. 74(6): 1516-1530.

[35] Incremental Cost-Effectiveness Ratio (ICER) [online]. (2016). York; York Health Economics Consortium; 2016. https://yhec.co.uk/glossary/incremental-cost-effectiveness-ratio-icer/

[36] Caulfield, J. (2019). How to do thematic analysis. *Retrieved on July, 7, 2020.*

[37] World Medical Association. Declaration of Helsinki, Fortaleza Brazil. Paris: World Medical Association; 2013.

[38] Bhambere, S, Abhishek, B, Sumit, H. Rapid digitization of healthcare—A review of COVID-19 impact on our health systems. Int. J. All Res. Educ. Sci. Methods. 2021. 9: 1457-1459.

[39] Mukherjee, D. Participation of older adults in virtual volunteering: A qualitative analysis. Ageing International. 2011. 36(2): 253-266.

Figure 1.

Schematic of patient enrolment and data collection in the ITV-PAL study. Own elaboration.

For peer review only

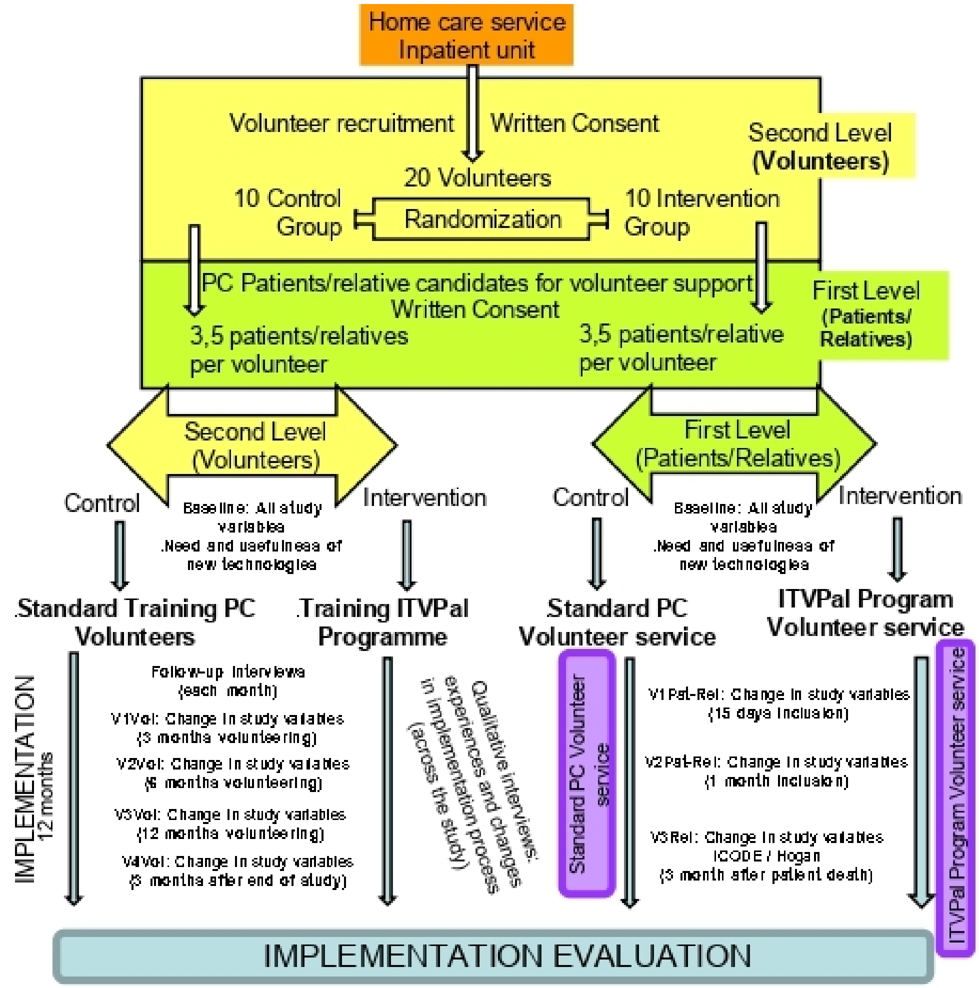

Figure 1. Schematic of patient enrolment and data collection in the ITV-PAL study: Own elaboration.

90x90mm (150 x 150 DPI)

BMJ Open

ITV-Pal Programme: Protocol of evaluation of the Implementation of Tech-Volunteer Programme in Palliative Care Services

Journal:	*BMJ Open*
Manuscript ID	bmjopen-2022-065222.R2
Article Type:	Protocol
Date Submitted by the Author:	24-Mar-2023
Complete List of Authors:	Barnestein-Fonseca, Pilar; CUDECA Foundation; IBIMA,<br>Víbora Martín, Eva; Universidad de Malaga, Department of Social Psychology; CUDECA Foundation<br>Ruiz-Torreras, Inmaculada; CUDECA Foundation; Universidad de Malaga, Department of Social Psychology<br>Chapinal-Bascón, Helena; CUDECA Foundation<br>Martín-Roselló, Maria ; CUDECA Foundation; IBIMA<br>Gómez García, Rafael; CUDECA Foundation; IBIMA
**Primary Subject Heading**:	Palliative care
Secondary Subject Heading:	Qualitative research
Keywords:	PALLIATIVE CARE, Telemedicine < BIOTECHNOLOGY & BIOINFORMATICS, COVID-19, PUBLIC HEALTH

SCHOLARONE™
Manuscripts

Title: *ITV-Pal Programme: Protocol of evaluation of the Implementation of Tech-Volunteer Programme in Palliative Care Services*

Authors: Pilar Barnestein-Fonseca[2,3] (pilarbarnestein@cudeca.org)
Eva Víbora-Martín [1,2,4] (evavibora@cudeca.org)
Inmaculada Ruiz-Torreras [1,2,4] (inmaruiz@cudeca.org)
Helena Chapinal-Bascón [1] (helenachapinal@cudeca.org)
María Luisa Martín-Roselló [2,4] (marisamartin@cudeca.org)
Rafael Gómez-García [2,4] (rafaelgomez@cudeca.org)

[1] Department of Social Psychology, Social Work, Social Anthropology and East Asian Studies. Faculty of Psychology and Speech Therapy. University of Malaga.
[2] CUDECA Institute for Training and Research in Palliative Care, CUDECA Hospice Foundation, Málaga, Spain.
[3] Instituto de Investigación Biomédica de Málaga-IBIMA Group C08: Pharma economy: Clinical and economic evaluation of medication and Palliative Care, Málaga, Spain.
[4] Instituto de Investigación Biomédica de Málaga-IBIMA Group CA15: Palliative Care, Málaga, Spain.

Correspondence to
Eva Víbora-Martín[1,2,4] (evavibora@cudeca.org)
[1] Department of Social Psychology, Social Work, Social Anthropology and East Asian Studies. Faculty of Psychology and Speech Therapy. University of Malaga.
[2] CUDECA Institute for Training and Research in Palliative Care, CUDECA Hospice Foundation, Málaga, Spain.
[4] Instituto de Investigación Biomédica de Málaga-IBIMA Group CA15: Palliative Care, Málaga, Spain.
+34 652 81 30 16

Abstract

Introduction: Distress at the end of life is significantly associated with physical/somatic and non-physical symptoms, but dying is also a social process. Volunteer support represents an important and necessary community resource. New technologies may become a useful resource in this kind of situation and have already proven a benefit in the assistance context of palliative care.

Although volunteers contribute millions of hours of work, the review of bibliography found no sufficiently robust studies to merit inclusion, and even less when associating palliative care volunteering and New Technologies.

As more research is needed on the impact of training and support for palliative care volunteers, we intend to study the state of art of technophilia and technophobia, and according to the results, implement and evaluate a volunteer training programme in the use of new technologies to support patients dying at home and their relatives.

Methods and analysis: A mixed-method study design will be conducted. We will recruit 20 volunteers and 70 patients in two years. Intervention: training of volunteers in new technologies and volunteer

For peer review only

accompaniment of patients/relatives using technologies. The control group will accompany patients as usual.

Ethics and dissemination: Ethics approval for the ITV-Pal Programme project was granted by the Malaga Regional Research Ethics Committee. As new knowledge is gained from this project, findings will be disseminated through publications, presentations and feedback to clinicians who are participating in this study.

Trial registration: NCT04900103

Keywords: New technologies, volunteering, palliative care, digital volunteering, mixed-method.

Article Summary

Strengths and limitations of this study

- The best way to evaluate the implementation of a service is to combine quantitative and qualitative methodology. Therefore, the ITV-PAL programme combines both methods. Quantitative methods are used to measure key process variables and allow testing of pre-hypothesised mechanisms of impact and contextual moderators. Qualitative methods help to capture emerging changes in implementation, experiences of the intervention and unanticipated or complex causal pathways, as well as to generate new theory.
- It may be that the effect of the intervention on recruitment results in different recruitment rates. For example, when randomising, a volunteer may not feel comfortable being assigned to the intervention group.
- The principal risk in this study is that we may not be able to attend to the different needs of patients/relatives. In this case, we will try to adapt by offering other volunteers who may be able to give a better response to these needs.

Introduction

How we care for the dying is perhaps the most pressing personal, social and public health issue of the 21st century. The experience of dying as an integral and acknowledged part of individual life, supported by the very best personalised care, is currently far from being standard practice in Europe and beyond [1]. Furthermore, it is estimated that by 2050, 1 in 5 people in the world will be over the age of 60 [1] with an estimated 52 million people expected to die worldwide in 2030 from chronic diseases such as cancer, cardiovascular and renal diseases and others [2]. This poses a new challenge to the functional capacity and organisational structures of current healthcare systems.

For peer review only

Distress at the end of life is significantly associated with both physical/somatic and non-physical symptoms. For this reason, patient care cannot focus only on treating physical symptoms through medication. When a terminal illness disrupts a life, it leads to the loss of important relationships, as well as changing the structure of the patient's social network and the levels of relationship and support [2]. Dying is therefore also a social process [3]. The psychological distress due to feelings of anxiety, depression, loneliness and being a burden requires additional approaches to care [4, 5]. Approaches that aim to provide supplementary, informal, lay support for patients dying at home, and their relatives, may introduce a valuable sense of 'community' and lead to important improvements in the care of the dying. Volunteer support represents an important and necessary community resource for patients and relatives to enhance the experience of living at the end of life and dying [4, 5]. Although few studies exist, those few agree on the beneficial aspects of volunteering on patient and family satisfaction at the end of life [4, 5].

It is of paramount importance to develop innovative, economically viable interventions to support the care that dying patients and their relatives receive. In this way, the current health emergency situation and the confinement has revealed the isolation and loneliness that certain groups have already experienced for a long time, such as the elderly, immigrants or patients and caregivers [6]. NT may become a useful resource in this kind of situation and have already proven a benefit in the context of palliative care (PC). Health care professionals (HCP) use information and communication technologies to facilitate health services for patients who have problems with access to the services, any disability which restricts movement or who live in a rural area [7, 8]. In some studies, they found that the use of new technologies (NT) in consultation was as effective as face-to-face [9-13]. It was even more positive for caregivers, reducing their anxiety and increasing their quality of life [14]. In this context, the implementation of services that combine volunteer support and NT can be an innovative, economically viable intervention to support the care of dying patients and their relatives. In addition, these approaches bring volunteering closer to people who would like to offer their help but have little time or cannot be in the same place as the patient/relatives. In these cases, virtual volunteering could be a way to reach a larger population [15].

Studies related to volunteering with NT mainly address the challenges of bringing these types of devices closer to the older population [16, 17] or how volunteers perceive them as another resource to improve their relationship with the caregivers of patients [18], but even so organisations are far from including such resources in their practice.

Technologies resources facilitate volunteer **interaction and communication** with the community, which is increasingly dependent on NT. Furthermore, volunteers with NT training will be able to help patients with **daily practical tasks.** NTs have also proven useful for **therapeutic interventions.** The

For peer review only

patient can use them to review their biography, maintain social relationships with people who live far away or help them to build a legacy for their loved ones. [19, 20]

Although volunteers contribute many hours to accompanying and supporting patients and their relatives, the review of bibliography found no sufficiently robust studies to merit inclusion, and even less when associating PC volunteering and NT. More research is needed to assess the impact of training and support of volunteers in PC. This study aims to analyse the technophobia (fear or aversion towards new technologies or complex devices) and technophilia (opposed to technophobia and is responsible for establishing the behaviour of adherence, often uncritically, to technological innovations) of patients, relatives, volunteers and health professionals. Once their knowledge of new NT has been assessed, a training programme for volunteers will be evaluated and a programme of accompaniment through technological devices will be implemented.

The **aim of this study will be to** evaluate the implementation of a volunteer training programme in the use of NT (specifically through smartphones and tablets) to support patients facing a life-threatening illness and their relatives. The specific objectives will be: 1) To explore the need and usefulness of NT from the point of view of patients, relatives, volunteers and HCP in PC and describe their technological profile; 2) To design a tech-volunteer curriculum and implement a tech-volunteer training programme; 3) To implement the tech-volunteer programme within a PC Home care service and Inpatient unit and assess its impact on the care provided.

Methodology:

The Research Ethical Committee of Málaga (25/02/2021) approved this study.

Study Design

The best approach to evaluate the implementation process is through mixed-method and the combination of quantitative and qualitative methods [21]. Basic quantitative measures of implementation may be combined with in-depth qualitative data to provide detailed understanding of intervention functioning on a small scale. Quantitative methods will measure key process variables and allow testing of pre-hypothesised mechanisms of impact and contextual moderators. Qualitative methods will capture emerging changes in implementation, experiences of the intervention and unanticipated or complex causal pathways, as well as generating a new theory.

A - Quantitative approach:

- **Pragmatic cluster randomised clinical trial** to test efficacy (the unit of randomization is the volunteer and the unit of analysis is the patient/relatives). A pragmatic cluster randomised controlled trial has shown to be the most appropriate design when the programmes are more focused on the organisational level [22,23]. For our study, the cluster design is based on two

levels: the higher or second level is represented by the volunteer (over volunteer the intervention is conducted, ITVPal Programme: Tech-Volunteer Programme in Palliative Care Services), and the lower or first level being represented by the patients/relatives, who have agreed to participate and are going to receive the tech-volunteer care from volunteers of ITVPal Programme.

- **Before-after design** for satisfaction of volunteers and HPC with the intervention and its implementation to test effectiveness. The design involves evaluating the effects of a deliberate intervention (introduction of ITVPal Programme) by comparing the outcomes of study participants investigated before the intervention with those measured afterwards.
- **Cost-utility study** from the perspective of the funder with a time horizon of two years. A detailed cost analysis including costs for the adaptation of a volunteer standard course to an ITVPal Programme as well as the recruitment and training costs of the volunteers will be performed.

<u>B - Qualitative approach:</u> **interviews** (individuals and groups) with HCP, volunteers and with key informants of patients/relatives to test at the beginning the need and usefulness of NT, and also during the implementation process to test changes and experiences. A narrative approach will be adopted, facilitating the production of 'rich' data regarding how participants make 'sense' of and engage with the ITVPal programme. These interviews will explore the thoughts and feelings engendered by access to this service, initial motivations, as well as thoughts and feelings about care delivery as a whole. Narrative interviewing enables the participants to tell their 'stories', prompted by an open question.

Settings

The study will be conducted in a Palliative Care Unit from both a Home Care and an Inpatient Unit of the CUDECA Foundation.

The Hospitalisation Unit is a small Hospice consisting of 10 rooms where patients at the end of life are cared for in cases of uncontrolled symptoms, for family respite or in their final days, among other situations. In this unit, volunteers come every afternoon to the rooms where they wish to offer accompaniment to patients and their relatives.

In addition, CUDECA foundation provides home care to more than 1,500 patients a year through 7 care teams made up of a doctor, nurse, psychologist and social worker. In these cases, the volunteer accompanies patients and relatives who wish them to do so.

Participants

A total of 140 patients and/or relatives will be selected from the Home Care and Inpatient Unit of CUDECA Foundation where the ITV-Pal program will be implemented. This sample size is enough to

For peer review only

detect a mean difference of 3 in the HAD scores between groups of 25%, with a power of 80% and a confidence level of 95% [24]. Sample size was adjusted according to the standard criteria for cluster randomised trials, using the design effect (DE) of 1.3. The DE was calculated as follows: DE = 1 + (nc – 1)*ICC (where nc is the mean number of individuals in the cluster and ICC the intra-cluster correlation coefficient). The ICC in the present work was considered to be 0.05 and the mean cluster size was assumed to be 7. A potential loss of 20% was estimated. Therefore, 140 patients/relatives and 20 volunteers are required.

The inclusion criteria will be: patients and relatives cared for by a specialised Palliative Care team; to be over 18 years of age; consenting to participate in the study by signing the informed consent form. Subjects who have specific conditions reducing their physical ability to use the devices, for example, visual, hearing, or motor impairments will be excluded from the study.

The HCP involved in these services will be included to measure the success of implementing the program.

The inclusion criteria for volunteers will be an interview with the Volunteer Department and having completed basic care training to be able to carry out their work. Also a commitment to participate in the study for one year. For this reason, more volunteers will be trained as needed during the two years of implementation.

Randomisation

The radomisation will take place in the higher or second level represented by the volunteer (over volunteer the intervention is conducted: ITVPal Programme) using a block randomisation technique. Each block is formed by four volunteers among whom the two study arms will be uniformly distributed. Once the blocks have been created, they will be distributed using a sequence of random numbers and the final list of volunteers allocations will be created. The final list of volunteers allocations will be guarded by the principal investigator of the project, and he will inform the allocation of each volunteers included in the trial.

Patient and Public Involvement

The design of this protocol did not involve patients.

Intervention

A complex intervention will be carried out in two phases.

- First phase: training of volunteers in NT. Volunteers will be trained in the use of NT and their interconnectivity as tools to support their work as volunteers.

The topics that will be covered in the volunteer training are the role of the volunteer, use of NT, communication strategies, complicated situations, self-care, summary and closure of the training. All of this is divided into 10 sessions, each lasting approximately 3 hours. This training will be integrated into the specific training programme for PC care volunteers.

The control group, on the other hand, will receive the standard training that the care volunteers have received so far.

- Second phase: volunteer accompaniment of patients/relatives. During the activities carried out by the volunteer with the patient for accompaniment and leisure. It will have the support of a technological device to be used according to the needs of the patient/relatives (e.g. internet searches on a topic of interest, video calls, phone calls, virtual visits, etc.) It will be an outcome of the study to know what the device has been used for.

The control group will accompany patients without using the NT as usual.

Variables

Sociodemographic variables such as date of birth, gender, educational level, nationality, spiritual beliefs and employment status will be assessed in all groups.

Specifically, relatives will be asked about their relationship with the patient. Volunteers will be questioned about their time of collaboration and whether they have had personal experience with palliative patients and finally, health professionals will be asked specifically about their work experience in PC.

In the following section, the different tools of assessment that each group will have to complete will be indicated:

Patients:

- ICECAP-SCM [25] has been developed as a tool for use in economic evaluation conducted in an end of life setting for patients. The questionnaire covers the attributes of: choice, love and affection, physical suffering, emotional suffering, dignity, being supported and preparation with the lowest score being 1 and the highest score being 4.
- EuroQol-5D-5L [26], a generic, brief and easy-to-use questionnaire designed to describe and

For peer review only

assess the current health-related quality of life of the person completing the questionnaire. Primary outcome. The descriptive system comprises five dimensions: mobility, self-care, usual activities, pain/discomfort and anxiety/depression. Each dimension has 5 levels: no problems, slight problems, moderate problems, severe problems and extreme problems. The patient is asked to indicate his/her health state by ticking the box next to the most appropriate statement in each of the five dimensions.

- Distress Thermometer [27] was developed as a simple tool to effectively screen for symptoms of distress. The instrument is a self-reported tool using a 0-to-10 rating scale.
- Edmonton Symptom Assessment Questionnaire [28] used to rate the intensity of nine common symptoms experienced by cancer patients, including pain, tiredness, nausea, depression, anxiety, drowsiness, appetite, well-being and shortness of breath where 0 is that the symptomatology does not affect him/her at the moment and 10 with the highest intensity.
- Tech-PH [29] an instrument for measuring older people's attitudes toward technology. It was created from the six items in the two factors, techEnthusiasm and techAnxiety. TechPH could be interpreted on a five-point response scale, ranging from 1 (fully disagree) to 5 (fully agree), where the higher the index indicates a higher level of technophilia.
- QFSAS [30] was designed to assess the frequency and degree of satisfaction with perceived social support from different sources in relation to three types of support: emotional, informational and instrumental. Both frequency and satisfaction are measured on a likert scale of 1 to 5.
- HADS [31] instrument to measure anxiety and depression in a general medical population of patients. The lowest score being 0 and the highest score being 3.

Relatives:

- ICECAP-CPM [32] has been developed as a tool for use in economic evaluation conducted in an end of life setting for relatives. The questionnaire covers the attributes of: good communication, privacy and space, emotional support, practical support, being able to prepare and cope and being free from emotional distress related to the condition of the decent with the lowest score being 1 and the highest score being 5.
- EuroQoL-5D-5L [26]
- Distress Thermometer [27]
- TechPH [29]

For peer review only

- QFSAS [30]
- HADS [31]
- iCODE [33] is a self-completion post-bereavement questionnaire, based on the key components of best practice in end of life care. It assesses quality of care for the dying through 32 main questions, reflecting core palliative care principles.

Volunteers:

- ICECAP-A [34] is a measure of capability for the general adult (18+) population for use in economic evaluation. The questionnaire covers the attributes of: attachment (an ability to have love, friendship and support), stability (an ability to feel settled and secure), achievement (an ability to achieve and progress in life), enjoyment (an ability to experience enjoyment and pleasure) and autonomy (an ability to be independent) with the lowest score being 1 and the highest score being 4.
- EuroQoL-5D-5L [26].
- Distress Thermometer [27].
- Satisfaction with volunteering; Motivation (VFI) [35] a 30 item measure of motivations to volunteer. Respondents answer each item on a 7-point scale ranging from 1 (not at all important/accurate) to 7 (extremely important/accurate).
- TechPH [29]

HCP:

- TechPH [29]

Focus groups or in-depth interviews will be conducted with all groups at different stages of the study to assess the implementation of the study.

Process Evaluation: Service Improvement Metrics: Process evaluation measures will be collected to assess the 'success' of the implementation process and how the service is being delivered. Data will be collected to assess the following: fidelity, dose and reach. Fidelity: Success of the Infrastructure. Dose: identification of patients, eligibility and take up of the service and whether the intervention was delivered as intended. Reach: (spread of implementation) quantifying the intervention implemented with the number of patients eligible for the service, number of patients who accept the service, number of wards with 'eligible' patients and number of wards where the service is implemented.

For peer review only

Data collection and follow up

The recruitment of the volunteers began in March 2021. When they finished their training, the new service started in September 2021 which was when patients/relatives recruitment started. Volunteer recruitment will be active during the whole study every three months. Recruitment is expected to end in September 2023.

The care teams offer the accompaniment of a volunteer to the patient/relatives. If they accept the accompaniment and participate in the study (if they want the accompaniment of a volunteer but do not want to participate in the study, the volunteer will be offered anyway). Once the informed consent to participate in the study has been signed, the data collection will proceed as described below. Further details can be seen in figure 1:

Patients/relatives who consent to participate (intervention and control group) will be invited to fill in at least 3 short questionnaires: at the time of recruitment for this study, 15 days after recruitment following the volunteer service experience (if hospitalised), and every month as long as the patient remains under PC care or until death. During the follow-up, patients and relatives will be asked if there is any change in their condition with respect to the questions asked at baseline. In this way, costly follow-up will be avoided. A maximum of 5 patients/relatives will also be invited to participate in an in-depth interview at various points during the implementation of the programme.

Volunteers will spend one year accompanying patients in ITV-Pal. In the following year, there will be other newly trained volunteers joining the project. During their time, they will fill in five questionnaires: baseline, 3, 6 and 12 months following volunteering, and 3 months after. A maximum of 5 volunteers and 5 HCP with direct experience of the service will be approached for inclusion in in-depth interviews to explore their experiences during the implementation process and at the end of this study.

Figure 1

Schematic of patient enrolment and data collection in the ITV-PAL study. Own elaboration.

Data analysis:

Quantitative data: Intention-to-treat principle. Descriptive statistics will be used to summarise subject characteristics (age, gender, education, religion, socioeconomic status, marital status, place of residence) and the process evaluation. Basal comparison between groups for independent samples: Student t-test or chi-square test; for related samples: ANOVA for repeated measures to analyse changes within groups. Multilevel analysis will be used for main and secondary outcomes. A multilevel

For peer review only

modelling approach will be used to examine the difference in the primary endpoint between patients/relatives, taking account of clustering effects at volunteers level. All statistical tests will be two sided and considered significant if $p < 0.05$. Repeated measurements of analyses of variance will be conducted to assess the development of endpoints over time. Multivariate Imputation will be used to handle missing data.

Also, outcome data will be collected for patients and relatives: the cost-effectiveness of care of dying patients, including the volunteer training programme, will be assessed by calculating the incremental cost-effectiveness ratio (ICER). The ICER is defined as the difference in total health care costs in the intervention group (including the costs of performing the intervention) compared to the total costs in the control group, divided by the difference in outcomes [36]. Outcomes will be measured also as QALYs using the EQ-5D health- related quality of life questionnaire. We will also use the capabilities approach (ICECAP). The potential impact of the programme beyond the trial period will be extrapolated using modelling techniques.

Qualitative data: Thematic Analysis [36], with an iterative approach to coding and analysis. For example, what was said by one participant may inform or shed light on the words of another participant. Analysis will focus on the "substance of the interview" to interpret "meanings and perceptions created and shared during a conversation. Analysis of the data will explore the ideas, assumptions or concepts underpinning what participants are saying. Words or phrases will be coded at latent and semantic level to 'label' meaning identified by those words/phrases. 'Themes' will then be developed from these codes around a 'central observation' or concept to reflect patterns of shared meaning [37]. For example, to capture "something important" about how these participants make sense of, and utilise, the ITVPal Programme within the context of end of life care.

Analysis will be conducted within a constructionist paradigm, which asserts that meaning and experience are socially produced. Stages of analysis will be iterative and cyclical rather than linear and analysis may move back and forth between stages. In summary, these principles are [36]:

- Familiarisation and immersion with the data: repeated reading of the interviews.
- Generation of initial codes: annotations of initial thoughts against items/categories of text.
- Creating themes from codes: The minor items/categories that have initially been identified from the text will then be interrogated to generate overall 'themes'. Engaging with relevant published literature (theoretical perspectives and relevant research) should further enhance the evolving interpretation.
- Reviewing themes: ensure all collated extracts of text form a coherent pattern, reorganising and refining themes as required.
- Defining and naming themes: to define the 'essence' of what a theme is about.

For peer review only

- Reporting the analysis: a narrative 'story' of the data to provide a concise, coherent, logical account.

Cost-utility ratio: this is an exploratory objective (for which a specific design has not been applied). The cost-utility ratio will be estimated by dividing the total cost by the sum of the potential gains expressed in QALYs.

Ethics and Dissemination

The study will be carried out according to the Good Clinical Practice guidelines of the Helsinki declaration [38]. Informed consent is obtained from each patient to take part in the study and for their clinical records to be reviewed. This project was approved by the corresponding ethics review board of the Malaga Provincial Research Ethics Committee on 25/02/2021.

The database is hosted on the secure REDCap platform at the University of Málaga, Spain. REDCap is an important tool for data access, linkages and mobilisation. On agreeing to participate in the project, clinics receive a random identification code, and the principal investigator and statistics team is blind to the coding.

All results and conclusions drawn from this project will be disseminated through local presentations, national or international meetings, academic publications and feedback to project participants.

In addition, as this project is funded by the La Caixa Foundation, the main results of the study will also be published for dissemination to the general public in different articles uploaded on its website.

Discussion

The coronavirus pandemic is changing the way we are interacting, launching us seemingly overnight from the real world into the virtual world, and seeking ways to address the challenges of social distance [39]. Face to face support of PC volunteers has temporarily been suspended, so creativity and innovative solutions are needed to continue providing this care and support. The use of information and communication technologies and, specifically, NT, allows us to rethink the way volunteers act by integrating these resources to create "tech-volunteers" that will attend to their needs in a different way and also support new needs that have emerged more clearly now due to social distance, with a broader reach [40]. The United Nations Online Volunteer Programme states: *"Online volunteering is fast, easy – and most of all, effective when skilled, passionate individuals join forces online with great organisations, everyone wins."* but no robust study has been carried out to support this statement. In this way, this study will bring the real evidence toward NT integration as a useful tool not only to facilitate communication between volunteers and patients/relatives, but also to turn NT into instruments that support daily living and enhance care, when in the hands of trained tech-volunteers.

Although this new volunteer programme will be implemented during the pandemic to analyse the

For peer review only

effects on patients and their relatives, we hope that training in NT will make it easier for PC volunteers to offer new skills and increase the satisfaction of the people they accompany.

The implementation of this study has some limitations that we will try to control for and take into account. For example, in pragmatic cluster randomised controlled trials, the first bias to take into account is the selection bias, as the randomisation is usually applied in the second level, before recruitment of the first-level participants, which is where the intervention impact is measured. However, there are strategies that minimise this bias. First, the selection and follow-up of patients will be carried out by an external researcher who will have no knowledge of volunteer randomisation. Second, the effect of the intervention over recruitment can lead to different recruitment indexes, depending on the groups, due to the possibility that volunteers will be allocated a group in which they do not feel comfortable (e.g a volunteer who does not like the NT -technophobe- and will be allocated the intervention group) could be less motivated to participate. This issue will be taken into account in the analysis and it will be a result of the implementation process. There could be measurement bias due to mistakes in recording of some variables or interviewer bias due to the administration of the questionnaires. To minimise these biases, interviewers will have been trained previously to ensure that visits will be as homogeneous as possible. A data collection booklet and a manual that explains how each variable is measured will be designed.

Another bias that has already been mentioned is the involvement of volunteers in the year they will collaborate in ITV-Pal. For this reason, more volunteers will be trained. But also the number of patients/volunteers needed to achieve a representative sample and draw conclusions will be difficult to achieve in the two years of piloting the study.

Maybe the principal risk in this study will be that we will not be able to attend to the different needs of patients/relatives. In this case, we will try to adapt by offering other volunteers who may be able to give a better response to this need.

Due to the fact that in this study we want to assess the implementation of an intervention programme, all these issues that we might find during the process could contribute to an improvement of the programme and would be useful for new research and the implementation of new interventions in the future.

Acknowledgements We would first of all like to thank Fundación La Caixa for supporting this project and for enabling us to make it a reality. We would also like to thank all the patients, relatives, professionals and volunteers who have participated in the beginning of this project and have allowed this protocol to become a reality.

Authors Contributions

EV-M, PB-F and IR-T were in charge of project conception and protocol writing. PB-F focused on the

For peer review only

methodological part and EV-M and IR-T on the other parts. MLM-R revised the protocol for its final version.

EV-M and PB-F have produced the final version of this article and it has been reviewed by the rest of them. All authors have read and approved the final version of the manuscript.

All authors (EV-M, PB-F, IR-T, MLM-R, RG-G and HC-B) have contributed to the development and review of the drafts that were used to submit this project and secure the grant.

Funding This work was supported by “la Caixa” Foundation (SR20-00841)

Competing interests None declared

ORCID iDs

Pilar Barnestein-Fonseca http://orcid.org/0000-0003-2767-8017

Eva Víbora-Martín http://orcid.org/0000-0003-2680-8019

Inmaculada Ruiz-Torreras https://orcid.org/0000-0001-7155-9627

Helena Chapinal-Bascón http://orcid.org/0000-0001-8783-964X

María Luisa Martín-Roselló https://orcid.org/0000-0002-2206-246X

Rafael Gómez-García https://orcid.org/0000-0003-3904-8733

For peer review only

References

[1] Dagli RJ, Sharma A. Polypharmacy: a global risk factor for elderly people. Journal of international oral health: JIOH. 2014, 6(6):i.

[2] Valencia, MIB. Envejecimiento de la población: un reto para la salud pública. Revista Colombiana de Anestesiología. 2012: 40(3), 192-194.

[3] Luxardo, N., Vindrola-Padrós, C. Through an intercultural lens: Grounding end of life interventions in "cultural difference" approaches. Revista M. Estudos sobre a morte, os mortos eo morrer. 2016, 1(1): 178-197.

[4] Baron, RS, Cutrona, CE, Hicklin, D, Russel DW, Lubaroff, DM. Social support and immune function among spouses of cáncer patients. En: Hewstone, M, Manstead, A, Stroebe, W (Eds.). The Blackwell reader in social psychology. Oxford: Blackwell; 1997.

[5] Block, EMMD, Casarett, DJ, Spence, C, Gozalo, P, Connor, SR, Teno TM. Got volunteers? Association of hospice use of volunteers with bereaved family members' overall rating of the quality of end-of-life care. J. Pain Symptom Manage. 2010, 39: 502-506.

[6] Herbst-Damm, KL, Kulik JA. Volunteer support, marital status, and the survival times of terminally ill patients. Health Psychol. 2005, 24: 225-229.

[7] Gómez, IFL, Silva, EAT, Correa, CT, Zuluaga, SV, Morales, MMR, Serna, CG, et al. Uso de las tecnologías de información y comunicación para el cuidado del binomio maternofetal: revisión de tema. Medicina U.P.B. 2016; 34: 138-147.

[8] Álvarez-Tobón, VA, Luna-Gómez, IF, Torres-Silva, EA, Higuita-Úsuga, A, Rivera-Mejía, PT. Tecnologías de Información y Comunicación (TIC) aplicadas en cuidados paliativos: revisión de tema. Psicooncología, 2018: 15(2), 345-360.

[9] Rogante, M, Giacomozzi, C, Grigioni, M, et al. Telemedicine in palliative care: a review of systematic reviews. Ann Ist Super Sanita, 2016; 52: 434–442.

[10] Head, BA, Schapmire, TJ, Zheng, Y. Telehealth in palliative care: a systematic review of patient-reported outcomes. J Hosp Palliat Nurs, 2017; 19: 130–139.

[11] Steindal, SA, Nes, AAG, Godskesen, TE, et al. Patients' experiences of telehealth in palliative home care: scoping review. J Med Internet Res, 2020; 22:e16218.

[12] Tricco, AC, Lillie, E, Zarin, W, et al. PRISMA extension for scoping reviews (PRISMA-ScR): checklist and explanation. Ann Intern Med, 2018; 169: 467–473.

[13] Wallace, A, Croucher, K, Quilgars, D, et al. Meeting the challenge: developing systematic reviewing in social policy. Policy Polit, 2004; 32: 455–470.

For peer review only

[14] Demiris, G, Parker Oliver, D, Wittenberg-Lyles, E, et al. A noninferiority trial of a problem-solving intervention for hospice caregivers: in person versus videophone. J Palliat Med, 2012; 15: 653–660.

[15] Butgereit, L. Seven characteristics of a successful virtual volunteering platform, 2011 IST-Africa Conference Proceedings, Gaborone, 2011: 1-8.

[16] Mukherjee, D, An Exploratory Study of Older Adults' Engagement with Virtual Volunteerism. Journal of Technology in Human Services. 2010; 28(3): 188-196.

[17] Mukherjee, D, Participation of Older Adults in Virtual Volunteering. Ageing Int. 2011; 36: 253-266.

[18] Campos-Romero, S, Herskovic, V, Fuentes, C, Abarca, E. Perceptions on Connecting Respite Care Volunteers and Caregivers. Int. J. Environ. Res. Public Health 2020, 17: 2911.

[19] Espindola, AV, Benincá, CRS, Scortegagna, SA, Secco, AC, Abreu, APM. Terapia da dignidade para adultos com câncer em cuidados paliativos: um relato de caso. Temas em Psicologia, 2017, 25(2), 733-747.

[20] Frávega, A, Carnino, MA, Dominguez, N, Bernal, C. Adultos Mayores y Nuevas Tecnologías: la superación de una brecha tecnológica. 2008, In III Congreso de Tecnología en Educación y Educación en Tecnología.

[21] Moore, G.F., Audrey, S., Barker, M., Bond, L., Bonel, C., Hardeman, W., Moore, L., O'Cathain, A., Tinati, T., Wight, D., Baird, J. Process evaluation of complex interventions: Medical Research Council guidance. BMJ: British Medical Journal, 2015. 350: p. h1258.

[22] Campbell, M.J. Cluster randomised trials. Med J Aust, 2019, 210, (4), 154-156

[23] Campbell, M.J., Hemming, K., Taljaard, M. The stepped wedge cluster randomised trial: what it is and when it should be used. Med J Aust, 2019, 210, (6), 253-254.

[24] Bernhard, J, Sullivan, M, Hürny, C, Coates, A.S., Rudenstam, C.M. Clinical relevance of single item quality of life indicators in cancer clinical trials. British journal of cancer, 2001. 84(9): 1156-1165.

[25] Sutton, E.J., Coast, J. Development of a supportive care measure for economic evaluation of end-of-life care using qualitative methods. Palliative medicine, 2014. 28(2): 151-157.

[26] EuroQoL group. EuroQol-a new facility for the measurement of health-related quality of life. Health Policity 1990; 16: 199-208.

[27] Roth, AJ, Kornblith, AB, Batel-Copel, L, Peabody, E, Scher, HI, Holland, JC. Rapid screening for psychologic distress in men with prostate carcinoma: a pilot study. Cancer: Interdisciplinary International Journal of the American Cancer Society, 1998, 82(10): 1904-1908.

[28] Bruera, E, Kuehn, N, Miller, MJ, Selmser, P, Macmillan, K. The Edmonton Symptom Assessment System (ESAS): a simple method for the assessment of palliative care patients. Journal of Palliative Care, 1991, 7: 6-9.

[29] Anderberg, P, Eivazzadeh, S, Berglund, JS. A Novel Instrument for Measuring Older People's Attitudes Towards Technology (TechPH): Development and Validation. Journal of Medical Internet Research, 2019, 21(5): e:13951.

For peer review only

[30] Hombrados-Mendieta, I, Gómez-Jacinto, L, Domínguez-Fuentes, JM, García-Leiva, P, Castro-Travé, M. Types of social support provided by parents, teachers and classmates during adolescence. Journal of Community Psychology, 2012. 40(6): 645–664.

[31] Zigmond, AS, Snaith RP. The hospital anxiety and depression scale. Acta psychiatrica scandinavica, 1983, 67(6):361-70.

[32] Canaway, A, Al-Janabi, H, Kinghorn, P, Bailey, C, Coast, J. Development of a measure (ICECAP-Close Person Measure) through qualitative methods to capture the benefits of end-of-life care to those close to the dying for use in economic evaluation. Palliative Medicine, 2017. 31(1): 53-62.

[33] Mayland, CR, Lees, C, Germain, A, et al. Caring for those who die at home: the use and validation of 'Care Of the Dying Evaluation' (CODE) with bereaved relatives. BMJ Support Palliative Care, 2014. 4: 167–174.

[34] Al-Janabi, H, Flynn, TN, Coast, J. Development of a self-report measure of capability wellbeing for adults: the ICECAP-A. Quality of life research. 2012. 21(1): 167-176.

[35] Clary, EG, Snyder, M, Ridge, RD, Copeland, J, Stukas, AA, Haugen, J, Miene, P. Understanding and assessing the motivations of volunteers: A functional approach. Journal of personality and social psychology. 1998. 74(6): 1516-1530.

[36] Incremental Cost-Effectiveness Ratio (ICER) [online]. (2016). York; York Health Economics Consortium; 2016. https://yhec.co.uk/glossary/incremental-cost-effectiveness-ratio-icer/

[37] Caulfield, J. (2019). How to do thematic analysis. *Retrieved on July, 7, 2020.*

[38] World Medical Association. Declaration of Helsinki, Fortaleza Brazil. Paris: World Medical Association; 2013.

[39] Bhambere, S, Abhishek, B, Sumit, H. Rapid digitization of healthcare—A review of COVID-19 impact on our health systems. Int. J. All Res. Educ. Sci. Methods. 2021. 9: 1457-1459.

[40] Mukherjee, D. Participation of older adults in virtual volunteering: A qualitative analysis. Ageing International. 2011. 36(2): 253-266.

Figure 1.

Schematic of patient enrolment and data collection in the ITV-PAL study. Own elaboration.

For peer review only

For peer review only

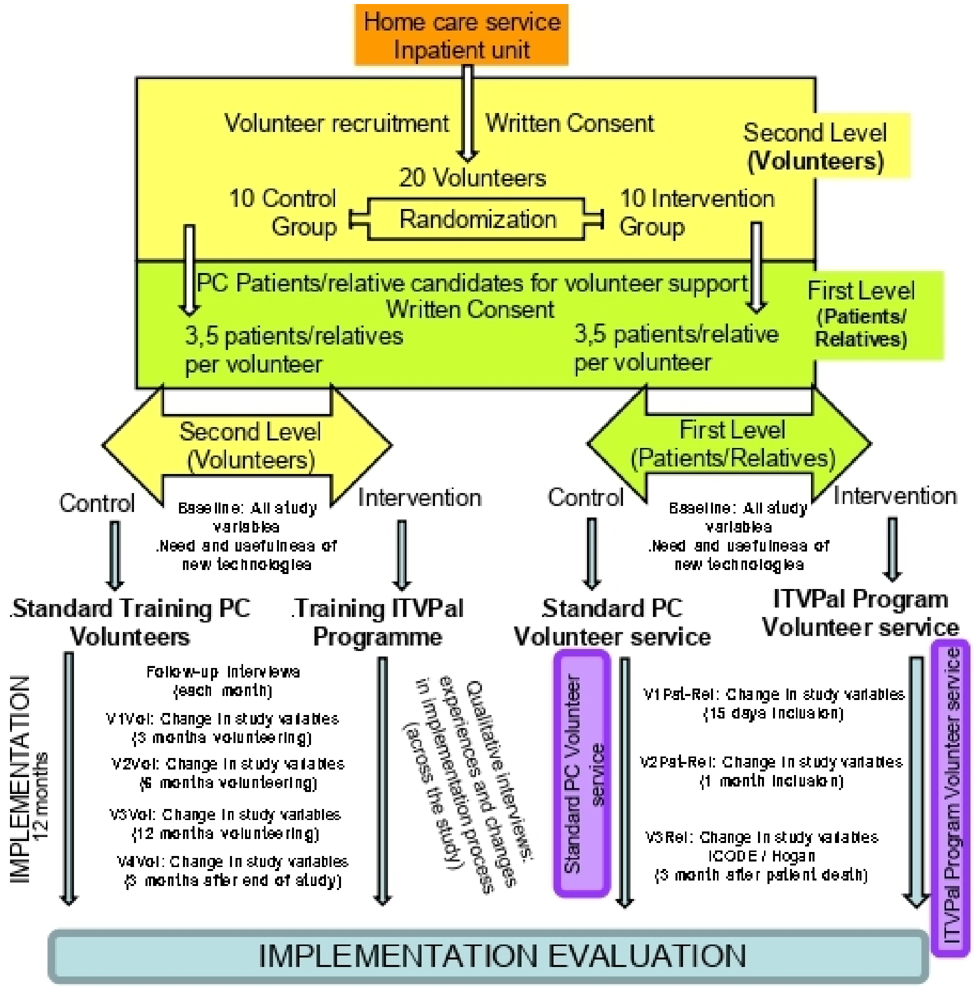

Figure 1. Schematic of patient enrolment and data collection in the ITV-PAL study: Own elaboration.

90x90mm (150 x 150 DPI)

BMJ Open

ITV-Pal Programme: Protocol of evaluation of the Implementation of Tech-Volunteer Programme in Palliative Care Services

Journal:	*BMJ Open*
Manuscript ID	bmjopen-2022-065222.R3
Article Type:	Protocol
Date Submitted by the Author:	30-Jun-2023
Complete List of Authors:	Barnestein-Fonseca, Pilar; CUDECA Foundation; IBIMA,<br>Víbora Martín, Eva; Universidad de Malaga, Department of Social Psychology; CUDECA Foundation<br>Ruiz-Torreras, Inmaculada; CUDECA Foundation; Universidad de Malaga, Department of Social Psychology<br>Chapinal-Bascón, Helena; CUDECA Foundation<br>Martín-Roselló, Maria ; CUDECA Foundation; IBIMA<br>Gómez García, Rafael; CUDECA Foundation; IBIMA
**Primary Subject Heading**:	Palliative care
Secondary Subject Heading:	Qualitative research
Keywords:	PALLIATIVE CARE, Telemedicine < BIOTECHNOLOGY & BIOINFORMATICS, COVID-19, PUBLIC HEALTH

SCHOLARONE™
Manuscripts

Title: *ITV-Pal Programme: Protocol of evaluation of the Implementation of Tech-Volunteer Programme in Palliative Care Services*

Authors: Pilar Barnestein-Fonseca[2 3] (pilarbarnestein@cudeca.org)
Eva Víbora-Martín [1 2 4] (evavibora@cudeca.org)
Inmaculada Ruiz-Torreras [1 2 4] (inmaruiz@cudeca.org)
Helena Chapinal-Bascón [1] (helenachapinal@cudeca.org)
María Luisa Martín-Roselló [2 4] (marisamartin@cudeca.org)
Rafael Gómez-García [2 4] (rafaelgomez@cudeca.org)

[1] Department of Social Psychology, Social Work, Social Anthropology and East Asian Studies. Faculty of Psychology and Speech Therapy. University of Malaga.
[2] CUDECA Institute for Training and Research in Palliative Care, CUDECA Hospice Foundation, Málaga, Spain.
[3] Instituto de Investigación Biomédica de Málaga-IBIMA Group C08: Pharma economy: Clinical and economic evaluation of medication and Palliative Care, Málaga, Spain.
[4] Instituto de Investigación Biomédica de Málaga-IBIMA Group CA15: Palliative Care, Málaga, Spain.

Correspondence to
Eva Víbora-Martín[1 2 4] (evavibora@cudeca.org)
[1] Department of Social Psychology, Social Work, Social Anthropology and East Asian Studies. Faculty of Psychology and Speech Therapy. University of Malaga.
[2] CUDECA Institute for Training and Research in Palliative Care, CUDECA Hospice Foundation, Málaga, Spain.
[4] Instituto de Investigación Biomédica de Málaga-IBIMA Group CA15: Palliative Care, Málaga, Spain.
+34 652 81 30 16

Abstract

Introduction: Volunteer support for patients and families at the end of life provides many benefits for the beneficiaries. New technologies could be a necessary resource in the accompaniment although, if there is little literature on palliative care volunteering in general, specifically on volunteering and new technologies, we find little information on the subject.

Therefore, the aim of this study is to implement and evaluate a training program for palliative care volunteers using new technologies in order to begin accompanying patients and families in hospital or at home.

Methods and analysis: A mixed-method study design will be conducted. We will recruit 20 volunteers and 70 patients in two years. Intervention: training of volunteers in new technologies and volunteer accompaniment of patients/relatives using technologies. The control group will accompany patients as usual.

Ethics and dissemination: Ethics approval for the ITV-Pal Programme project was granted by the Malaga Regional Research Ethics Committee. As new knowledge is gained from this project, findings

For peer review only

will be disseminated through publications, presentations and feedback to clinicians who are participating in this study.

Trial registration: NCT04900103

Keywords: New technologies, volunteering, palliative care, digital volunteering, mixed-method.

Article Summary

Strengths and limitations of this study

- The use of a mixed model that combines qualitative and quantitative methodology is the best way to evaluate the implementation of a new service, which is why it will be the one chosen in this study.
- One of the limitations of the study is the effect of the intervention on recruitment and that this may result in different recruitment rates.
- The main risk of this study is that we will not be able to attend to the different needs of the patients/families, although we will try to offer volunteers who can provide a better response to these needs.

Introduction

How we care for the dying is perhaps the most pressing personal, social and public health issue of the 21st century. The experience of dying as an integral and acknowledged part of individual life, supported by the very best personalised care, is currently far from being standard practice in Europe and beyond [1]. Furthermore, it is estimated that by 2050, 1 in 5 people in the world will be over the age of 60 [1] with an estimated 52 million people expected to die worldwide in 2030 from chronic diseases such as cancer, cardiovascular and renal diseases and others [2]. This poses a new challenge to the functional capacity and organisational structures of current healthcare systems.

Distress at the end of life is significantly associated with both physical/somatic and non-physical symptoms. For this reason, patient care cannot focus only on treating physical symptoms through medication. When a terminal illness disrupts a life, it leads to the loss of important relationships, as well as changing the structure of the patient's social network and the levels of relationship and support [2]. Dying is therefore also a social process [3]. The psychological distress due to feelings of anxiety, depression, loneliness and being a burden requires additional approaches to care [4, 5]. Approaches that aim to provide supplementary, informal, lay support for patients dying at home, and their relatives, may introduce a valuable sense of 'community' and lead to important improvements in the care of the dying. Volunteer support represents an important and necessary community resource for

For peer review only

patients and relatives to enhance the experience of living at the end of life and dying [4, 5]. Although few studies exist, those few agree on the beneficial aspects of volunteering on patient and family satisfaction at the end of life [4, 5].

It is of paramount importance to develop innovative, economically viable interventions to support the care that dying patients and their relatives receive. In this way, the current health emergency situation and the confinement has revealed the isolation and loneliness that certain groups have already experienced for a long time, such as the elderly, immigrants or patients and caregivers [6]. NT may become a useful resource in this kind of situation and have already proven a benefit in the context of palliative care (PC). Health care professionals (HCP) use information and communication technologies to facilitate health services for patients who have problems with access to the services, any disability which restricts movement or who live in a rural area [7, 8]. In some studies, they found that the use of new technologies (NT) in consultation was as effective as face-to-face [9-13]. It was even more positive for caregivers, reducing their anxiety and increasing their quality of life [14]. In this context, the implementation of services that combine volunteer support and NT can be an innovative, economically viable intervention to support the care of dying patients and their relatives. In addition, these approaches bring volunteering closer to people who would like to offer their help but have little time or cannot be in the same place as the patient/relatives. In these cases, virtual volunteering could be a way to reach a larger population [15].

Studies related to volunteering with NT mainly address the challenges of bringing these types of devices closer to the older population [16, 17] or how volunteers perceive them as another resource to improve their relationship with the caregivers of patients [18], but even so organisations are far from including such resources in their practice.

Technologies resources facilitate volunteer **interaction and communication** with the community, which is increasingly dependent on NT. Furthermore, volunteers with NT training will be able to help patients with **daily practical tasks.** NTs have also proven useful for **therapeutic interventions.** The patient can use them to review their biography, maintain social relationships with people who live far away or help them to build a legacy for their loved ones. [19, 20]

Although volunteers contribute many hours to accompanying and supporting patients and their relatives, the review of bibliography found no sufficiently robust studies to merit inclusion, and even less when associating PC volunteering and NT. More research is needed to assess the impact of training and support of volunteers in PC. This study aims to analyse the technophobia (fear or aversion towards new technologies or complex devices) and technophilia (opposed to technophobia and is responsible for establishing the behaviour of adherence, often uncritically, to technological innovations) of patients, relatives, volunteers and health professionals. Once their knowledge of new NT has been

For peer review only

assessed, a training programme for volunteers will be evaluated and a programme of accompaniment through technological devices will be implemented.

The **aim of this study will be to** evaluate the implementation of a volunteer training programme in the use of NT (specifically through smartphones and tablets) to support patients facing a life-threatening illness and their relatives. The specific objectives will be: 1) To explore the need and usefulness of NT from the point of view of patients, relatives, volunteers and HCP in PC and describe their technological profile; 2) To design a tech-volunteer curriculum and implement a tech-volunteer training programme; 3) To implement the tech-volunteer programme within a PC Home care service and Inpatient unit and assess its impact on the care provided.

Methodology:

The Research Ethical Committee of Málaga (25/02/2021) approved this study.

Study Design

The best approach to evaluate the implementation process is through mixed-method and the combination of quantitative and qualitative methods [21]**.** Basic quantitative measures of implementation may be combined with in-depth qualitative data to provide detailed understanding of intervention functioning on a small scale. Quantitative methods will measure key process variables and allow testing of pre-hypothesised mechanisms of impact and contextual moderators. Qualitative methods will capture emerging changes in implementation, experiences of the intervention and unanticipated or complex causal pathways, as well as generating a new theory.

A - Quantitative approach:

- **Pragmatic cluster randomised clinical trial** to test efficacy (the unit of randomization is the volunteer and the unit of analysis is the patient/relatives). A pragmatic cluster randomised controlled trial has shown to be the most appropriate design when the programmes are more focused on the organisational level [22,23]. For our study, the cluster design is based on two levels: the higher or second level is represented by the volunteer (over volunteer the intervention is conducted, ITVPal Programme: Tech-Volunteer Programme in Palliative Care Services), and the lower or first level being represented by the patients/relatives, who have agreed to participate and are going to receive the tech-volunteer care from volunteers of ITVPal Programme.
- **Before-after design** for satisfaction of volunteers and HPC with the intervention and its implementation to test effectiveness. The design involves evaluating the effects of a deliberate intervention (introduction of ITVPal Programme) by comparing the outcomes of study participants investigated before the intervention with those measured afterwards.
- **Cost-utility study** from the perspective of the funder with a time horizon of two years. A

For peer review only

detailed cost analysis including costs for the adaptation of a volunteer standard course to an ITVPal Programme as well as the recruitment and training costs of the volunteers will be performed.

<u>B - Qualitative approach:</u> **interviews** (individuals and groups) with HCP, volunteers and with key informants of patients/relatives to test at the beginning the need and usefulness of NT, and also during the implementation process to test changes and experiences. A narrative approach will be adopted, facilitating the production of 'rich' data regarding how participants make 'sense' of and engage with the ITVPal programme. These interviews will explore the thoughts and feelings engendered by access to this service, initial motivations, as well as thoughts and feelings about care delivery as a whole. Narrative interviewing enables the participants to tell their 'stories', prompted by an open question.

Settings

The study will be conducted in a Palliative Care Unit from both a Home Care and an Inpatient Unit of the CUDECA Foundation.

The Hospitalisation Unit is a small Hospice consisting of 10 rooms where patients at the end of life are cared for in cases of uncontrolled symptoms, for family respite or in their final days, among other situations. In this unit, volunteers come every afternoon to the rooms where they wish to offer accompaniment to patients and their relatives.

In addition, CUDECA foundation provides home care to more than 1,500 patients a year through 7 care teams made up of a doctor, nurse, psychologist and social worker. In these cases, the volunteer accompanies patients and relatives who wish them to do so.

Participants

A total of 140 patients and/or relatives will be selected from the Home Care and Inpatient Unit of CUDECA Foundation where the ITV-Pal program will be implemented. This sample size is enough to detect a mean difference of 3 in the HAD scores between groups of 25%, with a power of 80% and a confidence level of 95% [24]. Sample size was adjusted according to the standard criteria for cluster randomised trials, using the design effect (DE) of 1.3. The DE was calculated as follows: DE = 1 + (nc – 1)*ICC (where nc is the mean number of individuals in the cluster and ICC the intra-cluster correlation coefficient). The ICC in the present work was considered to be 0.05 and the mean cluster size was assumed to be 7. A potential loss of 20% was estimated. Therefore, 140 patients/relatives and 20 volunteers are required.

The inclusion criteria will be: patients and relatives cared for by a specialised Palliative Care team; to be over 18 years of age; consenting to participate in the study by signing the informed consent form. Subjects who have specific conditions reducing their physical ability to use the devices, for example,

For peer review only

visual, hearing, or motor impairments will be excluded from the study.

The HCP involved in these services will be included to measure the success of implementing the program.

The inclusion criteria for volunteers will be an interview with the Volunteer Department and having completed basic care training to be able to carry out their work. Also a commitment to participate in the study for one year. For this reason, more volunteers will be trained as needed during the two years of implementation.

Randomisation

The radomisation will take place in the higher or second level represented by the volunteer (over volunteer the intervention is conducted: ITVPal Programme) using a block randomisation technique. Each block is formed by four volunteers among whom the two study arms will be uniformly distributed. Once the blocks have been created, they will be distributed using a sequence of random numbers and the final list of volunteers allocations will be created. The final list of volunteers allocations will be guarded by the principal investigator of the project, and he will inform the allocation of each volunteers included in the trial.

Patient and Public Involvement

The design of this protocol did not involve patients.

Intervention

A complex intervention will be carried out in two phases.

- First phase: training of volunteers in NT. Volunteers will be trained in the use of NT and their interconnectivity as tools to support their work as volunteers.

The topics that will be covered in the volunteer training are the role of the volunteer, use of NT, communication strategies, complicated situations, self-care, summary and closure of the training. All of this is divided into 10 sessions, each lasting approximately 3 hours. This training will be integrated into the specific training programme for PC care volunteers.

The control group, on the other hand, will receive the standard training that the care volunteers have

For peer review only

received so far.

- Second phase: volunteer accompaniment of patients/relatives. During the activities carried out by the volunteer with the patient for accompaniment and leisure. It will have the support of a technological device to be used according to the needs of the patient/relatives (e.g. internet searches on a topic of interest, video calls, phone calls, virtual visits, etc.) It will be an outcome of the study to know what the device has been used for.

The control group will accompany patients without using the NT as usual.

Variables

Sociodemographic variables such as date of birth, gender, educational level, nationality, spiritual beliefs and employment status will be assessed in all groups.

Specifically, relatives will be asked about their relationship with the patient. Volunteers will be questioned about their time of collaboration and whether they have had personal experience with palliative patients and finally, health professionals will be asked specifically about their work experience in PC.

In the following section, the different tools of assessment that each group will have to complete will be indicated:

Patients:

- ICECAP-SCM [25] has been developed as a tool for use in economic evaluation conducted in an end of life setting for patients. The questionnaire covers the attributes of: choice, love and affection, physical suffering, emotional suffering, dignity, being supported and preparation with the lowest score being 1 and the highest score being 4.
- EuroQol-5D-5L [26], a generic, brief and easy-to-use questionnaire designed to describe and assess the current health-related quality of life of the person completing the questionnaire. Primary outcome. The descriptive system comprises five dimensions: mobility, self-care, usual activities, pain/discomfort and anxiety/depression. Each dimension has 5 levels: no problems, slight problems, moderate problems, severe problems and extreme problems. The patient is asked to indicate his/her health state by ticking the box next to the most appropriate statement in each of the five dimensions.
- Distress Thermometer [27] was developed as a simple tool to effectively screen for symptoms of distress. The instrument is a self-reported tool using a 0-to-10 rating scale.
- Edmonton Symptom Assessment Questionnaire [28] used to rate the intensity of nine common

For peer review only

symptoms experienced by cancer patients, including pain, tiredness, nausea, depression, anxiety, drowsiness, appetite, well-being and shortness of breath where 0 is that the symptomatology does not affect him/her at the moment and 10 with the highest intensity.

- Tech-PH [29] an instrument for measuring older people's attitudes toward technology. It was created from the six items in the two factors, techEnthusiasm and techAnxiety. TechPH could be interpreted on a five-point response scale, ranging from 1 (fully disagree) to 5 (fully agree), where the higher the index indicates a higher level of technophilia.
- QFSAS [30] was designed to assess the frequency and degree of satisfaction with perceived social support from different sources in relation to three types of support: emotional, informational and instrumental. Both frequency and satisfaction are measured on a likert scale of 1 to 5.
- HADS [31] instrument to measure anxiety and depression in a general medical population of patients. The lowest score being 0 and the highest score being 3.

Relatives:

- ICECAP-CPM [32] has been developed as a tool for use in economic evaluation conducted in an end of life setting for relatives. The questionnaire covers the attributes of: good communication, privacy and space, emotional support, practical support, being able to prepare and cope and being free from emotional distress related to the condition of the decent with the lowest score being 1 and the highest score being 5.
- EuroQoL-5D-5L [26]
- Distress Thermometer [27]
- TechPH [29]
- QFSAS [30]
- HADS [31]
- iCODE [33] is a self-completion post-bereavement questionnaire, based on the key components of best practice in end of life care. It assesses quality of care for the dying through 32 main questions, reflecting core palliative care principles.

Volunteers:

- ICECAP-A [34] is a measure of capability for the general adult (18+) population for use in economic evaluation. The questionnaire covers the attributes of: attachment (an ability to

For peer review only

have love, friendship and support), stability (an ability to feel settled and secure), achievement (an ability to achieve and progress in life), enjoyment (an ability to experience enjoyment and pleasure) and autonomy (an ability to be independent) with the lowest score being 1 and the highest score being 4.

- EuroQoL-5D-5L [26].
- Distress Thermometer [27].
- Satisfaction with volunteering; Motivation (VFI) [35] a 30 item measure of motivations to volunteer. Respondents answer each item on a 7-point scale ranging from 1 (not at all important/accurate) to 7 (extremely important/accurate).
- TechPH [29]

HCP:

- TechPH [29]

Focus groups or in-depth interviews will be conducted with all groups at different stages of the study to assess the implementation of the study.

Process Evaluation: Service Improvement Metrics: Process evaluation measures will be collected to assess the 'success' of the implementation process and how the service is being delivered. Data will be collected to assess the following: fidelity, dose and reach. Fidelity: Success of the Infrastructure. Dose: identification of patients, eligibility and take up of the service and whether the intervention was delivered as intended. Reach: (spread of implementation) quantifying the intervention implemented with the number of patients eligible for the service, number of patients who accept the service, number of wards with 'eligible' patients and number of wards where the service is implemented.

Data collection and follow up

The recruitment of the volunteers began in March 2021. When they finished their training, the new service started in September 2021 which was when patients/relatives recruitment started. Volunteer recruitment will be active during the whole study every three months. Recruitment is expected to end in September 2023.

The care teams offer the accompaniment of a volunteer to the patient/relatives. If they accept the accompaniment and participate in the study (if they want the accompaniment of a volunteer but do not want to participate in the study, the volunteer will be offered anyway). Once the informed consent to participate in the study has been signed, the data collection will proceed as described below. Further

For peer review only

details can be seen in figure 1:

Patients/relatives who consent to participate (intervention and control group) will be invited to fill in at least 3 short questionnaires: at the time of recruitment for this study, 15 days after recruitment following the volunteer service experience (if hospitalised), and every month as long as the patient remains under PC care or until death. During the follow-up, patients and relatives will be asked if there is any change in their condition with respect to the questions asked at baseline. In this way, costly follow-up will be avoided. A maximum of 5 patients/relatives will also be invited to participate in an in-depth interview at various points during the implementation of the programme.

Volunteers will spend one year accompanying patients in ITV-Pal. In the following year, there will be other newly trained volunteers joining the project. During their time, they will fill in five questionnaires: baseline, 3, 6 and 12 months following volunteering, and 3 months after. A maximum of 5 volunteers and 5 HCP with direct experience of the service will be approached for inclusion in in-depth interviews to explore their experiences during the implementation process and at the end of this study.

Figure 1

Schematic of patient enrolment and data collection in the ITV-PAL study. Own elaboration.

Data analysis:

Quantitative data: Intention-to-treat principle. Descriptive statistics will be used to summarise subject characteristics (age, gender, education, religion, socioeconomic status, marital status, place of residence) and the process evaluation. Basal comparison between groups for independent samples: Student t-test or chi-square test; for related samples: ANOVA for repeated measures to analyse changes within groups. Multilevel analysis will be used for main and secondary outcomes. A multilevel modelling approach will be used to examine the difference in the primary endpoint between patients/relatives, taking account of clustering effects at volunteers level. All statistical tests will be two sided and considered significant if $p < 0.05$. Repeated measurements of analyses of variance will be conducted to assess the development of endpoints over time. Multivariate Imputation will be used to handle missing data.

Also, outcome data will be collected for patients and relatives: the cost-effectiveness of care of dying patients, including the volunteer training programme, will be assessed by calculating the incremental cost-effectiveness ratio (ICER). The ICER is defined as the difference in total health care costs in the intervention group (including the costs of performing the intervention) compared to the total costs in

For peer review only

the control group, divided by the difference in outcomes [36]. Outcomes will be measured also as QALYs using the EQ-5D health- related quality of life questionnaire. We will also use the capabilities approach (ICECAP). The potential impact of the programme beyond the trial period will be extrapolated using modelling techniques.

Qualitative data: Thematic Analysis [36], with an iterative approach to coding and analysis. For example, what was said by one participant may inform or shed light on the words of another participant. Analysis will focus on the "substance of the interview" to interpret "meanings and perceptions created and shared during a conversation. Analysis of the data will explore the ideas, assumptions or concepts underpinning what participants are saying. Words or phrases will be coded at latent and semantic level to 'label' meaning identified by those words/phrases. 'Themes' will then be developed from these codes around a 'central observation' or concept to reflect patterns of shared meaning [37]. For example, to capture "something important" about how these participants make sense of, and utilise, the ITVPal Programme within the context of end of life care.

Analysis will be conducted within a constructionist paradigm, which asserts that meaning and experience are socially produced. Stages of analysis will be iterative and cyclical rather than linear and analysis may move back and forth between stages. In summary, these principles are [36]:

- Familiarisation and immersion with the data: repeated reading of the interviews.
- Generation of initial codes: annotations of initial thoughts against items/categories of text.
- Creating themes from codes: The minor items/categories that have initially been identified from the text will then be interrogated to generate overall 'themes'. Engaging with relevant published literature (theoretical perspectives and relevant research) should further enhance the evolving interpretation.
- Reviewing themes: ensure all collated extracts of text form a coherent pattern, reorganising and refining themes as required.
- Defining and naming themes: to define the 'essence' of what a theme is about.
- Reporting the analysis: a narrative 'story' of the data to provide a concise, coherent, logical account.

Cost-utility ratio: this is an exploratory objective (for which a specific design has not been applied). The cost-utility ratio will be estimated by dividing the total cost by the sum of the potential gains expressed in QALYs.

Ethics and Dissemination

The study will be carried out according to the Good Clinical Practice guidelines of the Helsinki declaration [38]. Informed consent is obtained from each patient to take part in the study and for their clinical

records to be reviewed. This project was approved by the corresponding ethics review board of the Malaga Provincial Research Ethics Committee on 25/02/2021.

The database is hosted on the secure REDCap platform at the University of Málaga, Spain. REDCap is an important tool for data access, linkages and mobilisation. On agreeing to participate in the project, clinics receive a random identification code, and the principal investigator and statistics team is blind to the coding.

All results and conclusions drawn from this project will be disseminated through local presentations, national or international meetings, academic publications and feedback to project participants.

In addition, as this project is funded by the La Caixa Foundation, the main results of the study will also be published for dissemination to the general public in different articles uploaded on its website.

Discussion

The coronavirus pandemic is changing the way we are interacting, launching us seemingly overnight from the real world into the virtual world, and seeking ways to address the challenges of social distance [39]. Face to face support of PC volunteers has temporarily been suspended, so creativity and innovative solutions are needed to continue providing this care and support. The use of information and communication technologies and, specifically, NT, allows us to rethink the way volunteers act by integrating these resources to create "tech-volunteers" that will attend to their needs in a different way and also support new needs that have emerged more clearly now due to social distance, with a broader reach [40]. The United Nations Online Volunteer Programme states: *"Online volunteering is fast, easy – and most of all, effective when skilled, passionate individuals join forces online with great organisations, everyone wins."* but no robust study has been carried out to support this statement. In this way, this study will bring the real evidence toward NT integration as a useful tool not only to facilitate communication between volunteers and patients/relatives, but also to turn NT into instruments that support daily living and enhance care, when in the hands of trained tech-volunteers.

Although this new volunteer programme will be implemented during the pandemic to analyse the effects on patients and their relatives, we hope that training in NT will make it easier for PC volunteers to offer new skills and increase the satisfaction of the people they accompany.

The implementation of this study has some limitations that we will try to control for and take into account. For example, in pragmatic cluster randomised controlled trials, the first bias to take into account is the selection bias, as the randomisation is usually applied in the second level, before recruitment of the first-level participants, which is where the intervention impact is measured. However, there are strategies that minimise this bias. First, the selection and follow-up of patients will be carried out by an external researcher who will have no knowledge of volunteer randomisation.

For peer review only

Second, the effect of the intervention over recruitment can lead to different recruitment indexes, depending on the groups, due to the possibility that volunteers will be allocated a group in which they do not feel comfortable (e.g a volunteer who does not like the NT -technophobe- and will be allocated the intervention group) could be less motivated to participate. This issue will be taken into account in the analysis and it will be a result of the implementation process. There could be measurement bias due to mistakes in recording of some variables or interviewer bias due to the administration of the questionnaires. To minimise these biases, interviewers will have been trained previously to ensure that visits will be as homogeneous as possible. A data collection booklet and a manual that explains how each variable is measured will be designed.

Another bias that has already been mentioned is the involvement of volunteers in the year they will collaborate in ITV-Pal. For this reason, more volunteers will be trained. But also the number of patients/volunteers needed to achieve a representative sample and draw conclusions will be difficult to achieve in the two years of piloting the study.

Maybe the principal risk in this study will be that we will not be able to attend to the different needs of patients/relatives. In this case, we will try to adapt by offering other volunteers who may be able to give a better response to this need.

Due to the fact that in this study we want to assess the implementation of an intervention programme, all these issues that we might find during the process could contribute to an improvement of the programme and would be useful for new research and the implementation of new interventions in the future.

Acknowledgements We would first of all like to thank Fundación La Caixa for supporting this project and for enabling us to make it a reality. We would also like to thank all the patients, relatives, professionals and volunteers who have participated in the beginning of this project and have allowed this protocol to become a reality.

Authors Contributions

EV-M, PB-F and IR-T were in charge of project conception and protocol writing. PB-F focused on the methodological part and EV-M and IR-T on the other parts. MLM-R revised the protocol for its final version.

EV-M and PB-F have produced the final version of this article and it has been reviewed by the rest of them. All authors have read and approved the final version of the manuscript.

All authors (EV-M, PB-F, IR-T, MLM-R, RG-G and HC-B) have contributed to the development and review of the drafts that were used to submit this project and secure the grant.

Funding This work was supported by “la Caixa” Foundation (SR20-00841)

Competing interests None declared

For peer review only

ORCID iDs

Pilar Barnestein-Fonseca http://orcid.org/0000-0003-2767-8017

Eva Víbora-Martín http://orcid.org/0000-0003-2680-8019

Inmaculada Ruiz-Torreras https://orcid.org/0000-0001-7155-9627

Helena Chapinal-Bascón http://orcid.org/0000-0001-8783-964X

María Luisa Martín-Roselló https://orcid.org/0000-0002-2206-246X

Rafael Gómez-García https://orcid.org/0000-0003-3904-8733

For peer review only

References

[1] Dagli RJ, Sharma A. Polypharmacy: a global risk factor for elderly people. Journal of international oral health: JIOH. 2014, 6(6):i.

[2] Valencia, MIB. Envejecimiento de la población: un reto para la salud pública. Revista Colombiana de Anestesiología. 2012: 40(3), 192-194.

[3] Luxardo, N., Vindrola-Padrós, C. Through an intercultural lens: Grounding end of life interventions in "cultural difference" approaches. Revista M. Estudos sobre a morte, os mortos eo morrer. 2016, 1(1): 178-197.

[4] Baron, RS, Cutrona, CE, Hicklin, D, Russel DW, Lubaroff, DM. Social support and immune function among spouses of cáncer patients. En: Hewstone, M, Manstead, A, Stroebe, W (Eds.). The Blackwell reader in social psychology. Oxford: Blackwell; 1997.

[5] Block, EMMD, Casarett, DJ, Spence, C, Gozalo, P, Connor, SR, Teno TM. Got volunteers? Association of hospice use of volunteers with bereaved family members' overall rating of the quality of end-of-life care. J. Pain Symptom Manage. 2010, 39: 502-506.

[6] Herbst-Damm, KL, Kulik JA. Volunteer support, marital status, and the survival times of terminally ill patients. Health Psychol. 2005, 24: 225-229.

[7] Gómez, IFL, Silva, EAT, Correa, CT, Zuluaga, SV, Morales, MMR, Serna, CG, et al. Uso de las tecnologías de información y comunicación para el cuidado del binomio maternofetal: revisión de tema. Medicina U.P.B. 2016; 34: 138-147.

[8] Álvarez-Tobón, VA, Luna-Gómez, IF, Torres-Silva, EA, Higuita-Úsuga, A, Rivera-Mejía, PT. Tecnologías de Información y Comunicación (TIC) aplicadas en cuidados paliativos: revisión de tema. Psicooncología, 2018: 15(2), 345-360.

[9] Rogante, M, Giacomozzi, C, Grigioni, M, et al. Telemedicine in palliative care: a review of systematic reviews. Ann Ist Super Sanita, 2016; 52: 434–442.

[10] Head, BA, Schapmire, TJ, Zheng, Y. Telehealth in palliative care: a systematic review of patient-reported outcomes. J Hosp Palliat Nurs, 2017; 19: 130–139.

[11] Steindal, SA, Nes, AAG, Godskesen, TE, et al. Patients' experiences of telehealth in palliative home care: scoping review. J Med Internet Res, 2020; 22:e16218.

[12] Tricco, AC, Lillie, E, Zarin, W, et al. PRISMA extension for scoping reviews (PRISMA-ScR): checklist and explanation. Ann Intern Med, 2018; 169: 467–473.

[13] Wallace, A, Croucher, K, Quilgars, D, et al. Meeting the challenge: developing systematic reviewing in social policy. Policy Polit, 2004; 32: 455–470.

For peer review only

[14] Demiris, G, Parker Oliver, D, Wittenberg-Lyles, E, et al. A noninferiority trial of a problem-solving intervention for hospice caregivers: in person versus videophone. J Palliat Med, 2012; 15: 653–660.

[15] Butgereit, L. Seven characteristics of a successful virtual volunteering platform, 2011 IST-Africa Conference Proceedings, Gaborone, 2011: 1-8.

[16] Mukherjee, D, An Exploratory Study of Older Adults' Engagement with Virtual Volunteerism. Journal of Technology in Human Services. 2010; 28(3): 188-196.

[17] Mukherjee, D, Participation of Older Adults in Virtual Volunteering. Ageing Int. 2011; 36: 253-266.

[18] Campos-Romero, S, Herskovic, V, Fuentes, C, Abarca, E. Perceptions on Connecting Respite Care Volunteers and Caregivers. Int. J. Environ. Res. Public Health 2020, 17: 2911.

[19] Espindola, AV, Benincá, CRS, Scortegagna, SA, Secco, AC, Abreu, APM. Terapia da dignidade para adultos com câncer em cuidados paliativos: um relato de caso. Temas em Psicologia, 2017, 25(2), 733-747.

[20] Frávega, A, Carnino, MA, Dominguez, N, Bernal, C. Adultos Mayores y Nuevas Tecnologías: la superación de una brecha tecnológica. 2008, In III Congreso de Tecnología en Educación y Educación en Tecnología.

[21] Moore, G.F., Audrey, S., Barker, M., Bond, L., Bonel, C., Hardeman, W., Moore, L., O'Cathain, A., Tinati, T., Wight, D., Baird, J. Process evaluation of complex interventions: Medical Research Council guidance. BMJ: British Medical Journal, 2015. 350: p. h1258.

[22] Campbell, M.J. Cluster randomised trials. Med J Aust, 2019, 210, (4), 154-156

[23] Campbell, M.J., Hemming, K., Taljaard, M. The stepped wedge cluster randomised trial: what it is and when it should be used. Med J Aust, 2019, 210, (6), 253-254.

[24] Bernhard, J, Sullivan, M, Hürny, C, Coates, A.S., Rudenstam, C.M. Clinical relevance of single item quality of life indicators in cancer clinical trials. British journal of cancer, 2001. 84(9): 1156-1165.

[25] Sutton, E.J., Coast, J. Development of a supportive care measure for economic evaluation of end-of-life care using qualitative methods. Palliative medicine, 2014. 28(2): 151-157.

[26] EuroQoL group. EuroQol-a new facility for the measurement of health-related quality of life. Health Policity 1990; 16: 199-208.

[27] Roth, AJ, Kornblith, AB, Batel-Copel, L, Peabody, E, Scher, HI, Holland, JC. Rapid screening for psychologic distress in men with prostate carcinoma: a pilot study. Cancer: Interdisciplinary International Journal of the American Cancer Society, 1998, 82(10): 1904-1908.

[28] Bruera, E, Kuehn, N, Miller, MJ, Selmser, P, Macmillan, K. The Edmonton Symptom Assessment System (ESAS): a simple method for the assessment of palliative care patients. Journal of Palliative Care, 1991, 7: 6-9.

[29] Anderberg, P, Eivazzadeh, S, Berglund, JS. A Novel Instrument for Measuring Older People's Attitudes Towards Technology (TechPH): Development and Validation. Journal of Medical Internet Research, 2019, 21(5): e:13951.

For peer review only

[30] Hombrados-Mendieta, I, Gómez-Jacinto, L, Domínguez-Fuentes, JM, García-Leiva, P, Castro-Travé, M. Types of social support provided by parents, teachers and classmates during adolescence. Journal of Community Psychology, 2012. 40(6): 645–664.

[31] Zigmond, AS, Snaith RP. The hospital anxiety and depression scale. Acta psychiatrica scandinavica, 1983, 67(6):361-70.

[32] Canaway, A, Al-Janabi, H, Kinghorn, P, Bailey, C, Coast, J. Development of a measure (ICECAP-Close Person Measure) through qualitative methods to capture the benefits of end-of-life care to those close to the dying for use in economic evaluation. Palliative Medicine, 2017. 31(1): 53-62.

[33] Mayland, CR, Lees, C, Germain, A, et al. Caring for those who die at home: the use and validation of 'Care Of the Dying Evaluation' (CODE) with bereaved relatives. BMJ Support Palliative Care, 2014. 4: 167–174.

[34] Al-Janabi, H, Flynn, TN, Coast, J. Development of a self-report measure of capability wellbeing for adults: the ICECAP-A. Quality of life research. 2012. 21(1): 167-176.

[35] Clary, EG, Snyder, M, Ridge, RD, Copeland, J, Stukas, AA, Haugen, J, Miene, P. Understanding and assessing the motivations of volunteers: A functional approach. Journal of personality and social psychology. 1998. 74(6): 1516-1530.

[36] Incremental Cost-Effectiveness Ratio (ICER) [online]. (2016). York; York Health Economics Consortium; 2016. https://yhec.co.uk/glossary/incremental-cost-effectiveness-ratio-icer/

[37] Caulfield, J. (2019). How to do thematic analysis. *Retrieved on July, 7, 2020.*

[38] World Medical Association. Declaration of Helsinki, Fortaleza Brazil. Paris: World Medical Association; 2013.

[39] Bhambere, S, Abhishek, B, Sumit, H. Rapid digitization of healthcare—A review of COVID-19 impact on our health systems. Int. J. All Res. Educ. Sci. Methods. 2021. 9: 1457-1459.

[40] Mukherjee, D. Participation of older adults in virtual volunteering: A qualitative analysis. Ageing International. 2011. 36(2): 253-266.

Figure 1.

Schematic of patient enrolment and data collection in the ITV-PAL study. Own elaboration.

For peer review only

For peer review only

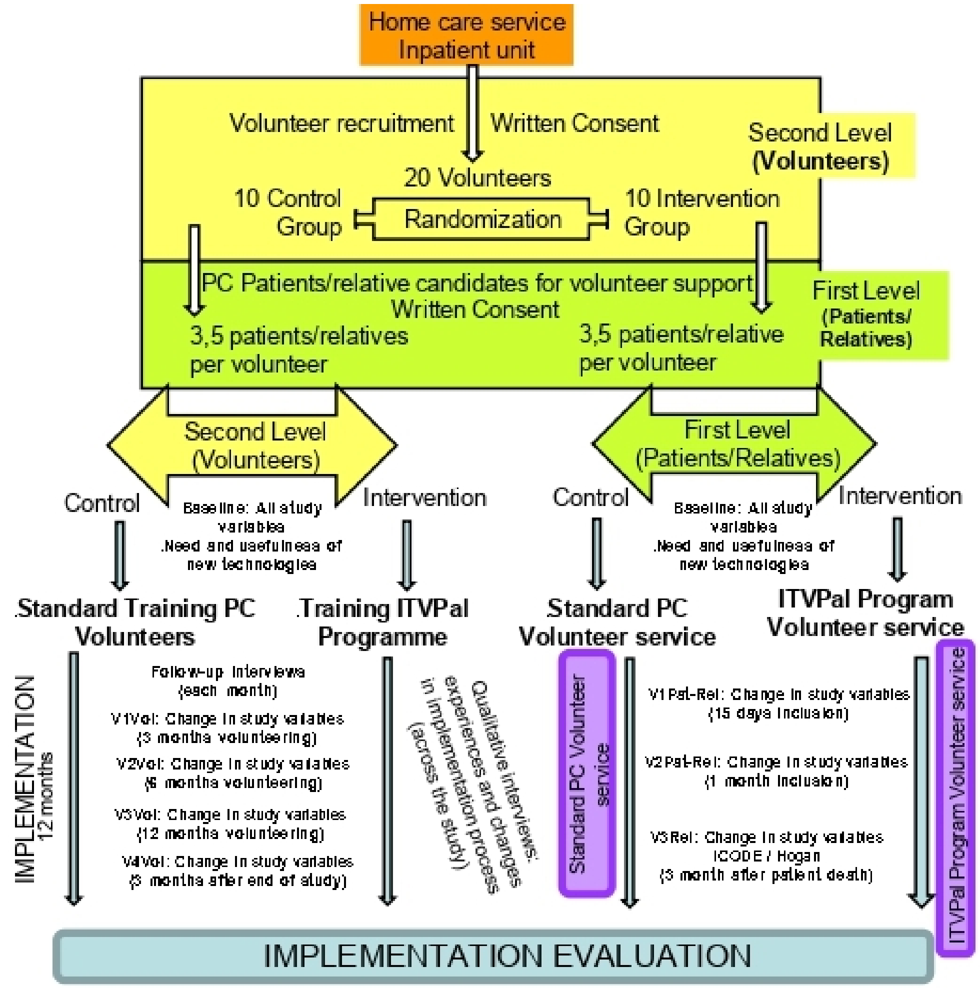

Figure 1. Schematic of patient enrolment and data collection in the ITV-PAL study: Own elaboration.

90x90mm (150 x 150 DPI)
